# CO Adsorption on Pd Nanoparticles: Assignment of Experimental C–O Vibrational Frequencies by DFT Calculations

**DOI:** 10.1021/acs.jpcc.5c08124

**Published:** 2026-02-05

**Authors:** Ilya V. Yudanov, Svetlana S. Laletina, Konstantin M. Neyman

**Affiliations:** † Institute of Solid State Chemistry and Mechanochemistry (ISSCMC) of the Siberian Branch of the Russian Academy of Sciences (SB RAS), 630128 Novosibirsk, Russia; ‡ Boreskov Institute of Catalysis (BIC), SB RAS, 630090 Novosibirsk, Russia; # Institute of Chemistry and Chemical Technology (ICCT), SB RAS, 660036 Krasnoyarsk, Russia; § Departament de Ciència de Materials i Química Física and Institut de Quimica Teòrica i Computacional, 16724 Universitat de Barcelona, c/Martí i Franquès 1, 08028 Barcelona, Spain; ∥ ICREA (Institució Catalana de Recerca i Estudis Avançats), Pg. Companys 23, 08010 Barcelona, Spain

## Abstract

Adsorption of CO probe molecules on metal catalysts is widely used to characterize the surface reactivity and morphology of these nanomaterials by assigning measured C–O vibrational frequencies to particular surface sites. Density-functional calculations of the corresponding CO adsorption complexes provide key complementary data for such characterization. However, even for the adequate structural models, the calculated frequencies do not quantitatively match the experimental values due to approximations in conventional generalized-gradient exchange–correlation functionals. We proposed a frequency-dependent scaling of the density-functional C–O frequencies for adsorption on different sites of nanostructured Pd catalysts, enabling quantitative agreement with the reference experimental values. Then, we computationally studied coverage-dependent bridge CO adsorption on edge sites of Pd nanoparticles, which revealed the energetic feasibility of the full CO occupation of these sites. Due to the static and dynamic CO–CO interactions, the calculated C–O stretching frequency grows by as much as 100 cm^–1^ from the singleton CO adsorbed value with the number of coadsorbates at the neighboring bridge-edge sites. The saturation frequency approaches 1990 cm^–1^, quantitatively matching the value experimentally observed for moderately large Pd particles. Using our frequency scaling, such particles are estimated to be at least 3 nm large.

## Graphical abstract

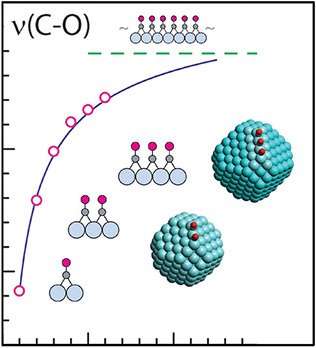

## Introduction

Metal nanoparticles (NPs) are of high interest for modern chemistry and chemical engineering, including catalytic applications, where Pd is one of the key catalytic metals.^1,2^ The knowledge of the relation between the size-dependent NP morphology and chemical activity is crucial for improving the catalytic performance. Much of the fundamental understanding of how the size and structure of a metal NP affect its properties originates from studies of well-defined model catalysts.^3–5^ Carbon monoxide, CO, being involved in many processes of environmental and energy catalysis, is also widely used as a probe to characterize the surface structure of metal NPs,^6–10^ including bimetallic nanosystems.^11–14^ A combination of experimental work on model nanosized catalysts with density-functional theory (DFT) calculations of model NPs was shown especially efficient for the assignment of observed spectroscopic features to particular structures at the atomic level.^15^

CO adsorption on metals is site-specific and is reflected by vibrational spectroscopy, where a distinct infrared (IR) band corresponds to each adsorption mode: on-top, bridge, or 3-fold hollow, where the CO molecule forms adsorption complexes with one, two, and three metal centers, respectively. Two bands with wavenumbers 1980–1990 and 2080–2090 cm^–1^ are typical for Pd NPs corresponding to bridge and on-top adsorbed CO, respectively.^6–8^ The peak positions for a given adsorption mode may vary with the surface morphology, e.g., bridge sites on facets and edges of Pd NPs differ in the adsorption energy and the frequency of C–O vibration.^15^ However, these differences of 10–20 cm^–1^ ^15^ are small compared to the frequency changes due to the interaction between coadsorbed CO species at the growing adsorbate coverage, even if the adsorption mode remains the same. For instance, on Pd(001), where only the 2-fold adsorption on bridge sites is observed in the wide range of coverages, the frequency of CO vibrations grows by 100 cm^–1^ from about 1890 cm^–1^ at the lowest coverage to about 1990 cm^–1^ at the saturation.^16,17^ This effect is now well studied, both experimentally and theoretically, for single-crystal surfaces Pd(001) and Pd(111).^18–22^ However, the effect is less understood for NPs since experimental studies deal mainly with samples saturated by CO, while DFT studies on model NPs usually consider a small number of CO adsorbates corresponding to low coverages. In particular, in our early DFT work, only a single adsorbed CO molecule per edge was located on model NPs, which resulted in the CO vibrational frequency being much lower than that observed experimentally.^15^ To fill this gap, in the present work, we studied how adsorption properties, including vibrational frequency, develop with increasing the number of CO molecules coadsorbed on edges of model Pd NPs. Unlike single-crystal (slab) studies, we consider finite numbers of coadsorbed CO molecules that enable estimating a minimal size of NPs, where the experimentally observed frequency can be reproduced.

The main goal of the manuscript is to quantify how the frequency of C–O vibration depends on the number of CO molecules coadsorbed at the edges of model Pd NPs. However, there is a problem that hinders direct comparison of experimental and DFT-calculated spectral data. It is the overestimated adsorption interaction that leads to underestimated computed vibrational frequencies when using the generalized-gradient approximation (GGA). This problem can be overcome by applying properly chosen site- and frequency-dependent scaling factors. We propose such suitable and accurate scaling factors prior to a detailed discussion of the calculated results on CO adsorption at the edges of Pd NPs.

## Computational Details

### Methods

The quantum chemical calculations were performed within the density-functional approach using the plane-wave VASP code.^23,24^ The interaction between nuclei and electrons was described by the projector augmented wave method.^25,26^ The plane-wave kinetic energy cutoff was set to 415 eV. The exchange and correlation effects were treated within the GGA using the Perdew–Burke–Ernzerhof (PBE) functional.^27^ Brillouin zone sampling was performed using the Monkhorst–Pack *k*-point grid.^28^ The Methfessel–Paxton smearing method^29^ of first order was employed to determine the electron occupancies with a smearing width of 0.1 eV.

Model NPs, bare and with CO adsorbate, were placed in a 2.5 × 2.5 × 2.5 nm^3^ periodic cell. The Brillouin zone in the calculations of finite-size NPs was sampled at the Γ-point only. In slab-model calculations, the surface Brillouin zone was sampled with a 5 × 5 × 1 *k*-point grid. All adsorption complexes on the metal surface were calculated as closed-shell electronic structures. The PBE-calculated lattice parameter of Pd bulk, 393.9 pm, was used for construction of slab models, where four bottom layers were fixed, and the rest relaxed prior to CO adsorption. The model NPs were fully relaxed prior to CO adsorption (note that due to the surface stress, the NPs of 200–300 Pd atoms have the lattice structure compressed by about 2% on average compared to the bulk metal). Positions of atoms in two surface layers of the metal substrates were optimized, along with the adsorbed species in calculations of the adsorption complexes. The optimization was considered complete when the forces acting on all relaxing atoms became smaller than 0.1 eV/nm. To calculate harmonic frequencies of C–O vibrations, the Hessian matrix was constructed using the finite difference method with the displacements of C and O atoms by ±1.5 pm from their equilibrium positions, keeping all metal atoms fixed. The Pd atom is heavy enough to neglect the effect of its motion on the frequency of the C–O stretching vibration. Our test calculations for a single CO molecule on the bridge-edge site of Pd_201_ NP (see Pd_201_ NP with two CO species in Figure 1) showed that if the displacements of two Pd atoms forming the bridge site are taken into account by the computational procedure, the frequency of C–O vibration changes by less than 1 cm^–1^, from 1847.6 to 1848.5 cm^–1^.

**Figure 1:**
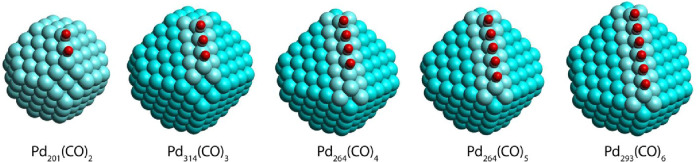
Studied models of Pd NPs with multiple CO molecules adsorbed with high density at the bridge sites of an NP edge. Pd atoms, positions of which were relaxed together with the adsorbed species, are shown in light blue.

Average adsorption energies per adsorbed CO molecule, *E*_ads_, were determined using the total energies of the adsorption complex, *E*(*n*CO/substrate), of the relaxed bare Pd substrate, *E*(substrate), and of the gas-phase CO, *E*(CO), according to the following expression:

$$E_{\mathrm{ads}}=(E(n\mathrm{CO}/\mathrm{substrate})-n\times E(\mathrm{CO})-E(\mathrm{substrate}))/n$$ 1

where *n* is the number of CO molecules in the unit cell. With this definition, negative *E*_ads_ corresponds to stable adsorption complexes on the surface.

### Nanoparticle Models

Our modeling approach based on DFT calculations of three-dimensional nanosized particles was developed to reliably simulate the surface properties of experimentally studied nanosized model catalysts with a well-ordered structure.^15,30–33^ Figure 1 shows the studied model NPs forming at their edge adsorption complexes with ensembles of CO molecules. Pd_201_ and Pd_314_ are truncated octahedrons with O_h_ symmetry. The models with longer edges, Pd_264_ and Pd_293_, with C_2v_ symmetry, were obtained from the O_h_ NPs Pd_314_ and Pd_405_, respectively, removing layers from two neighboring (111) facets forming the edge to be probed by CO adsorption. The resulting Pd_264_ and Pd_293_ NPs exhibit the edge length of six (6Pd) and seven Pd (7Pd) atoms, which can accommodate up to five and six bridge-adsorbed CO molecules, respectively (see Pd_264_(CO)_5_ and Pd_293_(CO)_6_ in Figure 1).

These NPs of 200–300 Pd atoms are larger than particles in the size region, where a strong increase of activity with transition to small clusters takes place,^34–37^ and, therefore, are suitable to reproduce the adsorption properties of even larger NPs, which are common to experimental studies of model catalysts.^3–9,11^

### Infinite Edge Model

As seen in Figure 1, the model Pd NPs exhibit finite size facets, which, by their structure and orientation, correspond to single-crystal (001) and (111) surfaces. In turn, the edges terminating two neighboring (111) facets (populated by CO in Figure 1) correspond in their orientation to the ridges on the single-crystal (110) surface. Therefore, to study the case of infinite edge terminating two (111) facets, we constructed slab models with unit cells comprising 68 and 102 Pd atoms (see the structure of the latter cell in Figure 2) derived from nine-layer slab models of the Pd(110) surface by removal of one out of the two [11̅0] rows from the top layer. Only half of the remaining ridge rows in these missing-row structures were populated by CO to exclude the interaction between adsorbates at the neighboring ridges. The smaller and the larger slabs contain two and three Pd atoms per ridge, providing, respectively, two and three bridge sites for CO adsorption. Thus, besides the full occupation, 1.0, of a selected ridge by CO illustrated in Figure 2, the lower CO occupations of 0.5 and 0.33 along the ridge were considered on smaller (68 atoms) and bigger (102) models, respectively.

**Figure 2:**
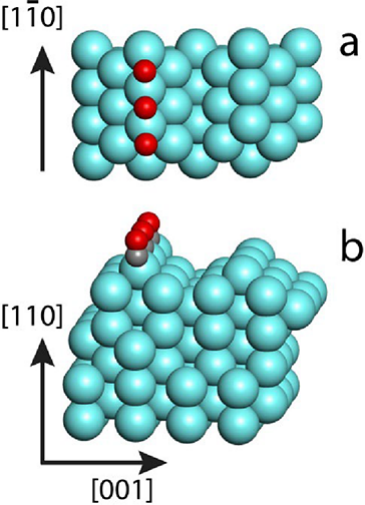
Periodic slab model of an infinite edge terminating two (111) planes—top (a) and side (b) views. The missing-row structure (unit cell of 102 Pd atoms) is constructed from the nine-layer ideal model of the (110) surface by removal of half of the surface rows.

## Results and Discussion

### Calibration of Calculated CO Vibrational Frequencies

Calculated DFT-GGA vibrational frequencies are commonly lower than the experimental ones. For the gas-phase CO molecule, the PBE exchange–correlation functional yields a harmonic frequency of 2125 cm^–1^, while the experimental frequency is 2143 cm^–1^. Accounting for the anharmonic contribution, the harmonic experimental frequency is 2169 cm^–1^.^38^ Scaling factors, *k*, are often used to establish the correspondence between calculated and experimental vibrational frequencies:

$$\nu_{\exp}=k\nu_{\mathrm{DFT}}$$ 2

In calculations of CO adsorption, it is also convenient to include the anharmonic effect in the scaling procedure: the scaling factor of 1.0085 transforms the calculated PBE harmonic frequency for gas-phase CO (2125 cm^–1^) to the experimental anharmonic value of 2143 cm^–1^.

In adsorption complexes formed by CO on metal surfaces, the so-called backdonation^39,40^ of electron density from metal to the antibonding vacant 2π* orbital of CO leads to weakening of the C–O bond and causes red shift (to the lower wavenumbers) of the C–O stretching frequency and elongation of the C–O bond length in the adsorbed state compared to the gas phase. C–O frequency for chemisorbed CO on Pd has been observed from about 2100 to 1800 cm^–1^, decreasing as the coordination of the adsorbed molecule by the surface sites increases. Figure 3 shows the frequency bands typical of different adsorption modes of CO on Pd substrates. In DFT-GGA calculations, the adsorption interaction is overestimated due to excessive backdonation manifested by an additional shift of the C–O stretching frequency to lower wavenumbers.

**Figure 3:**
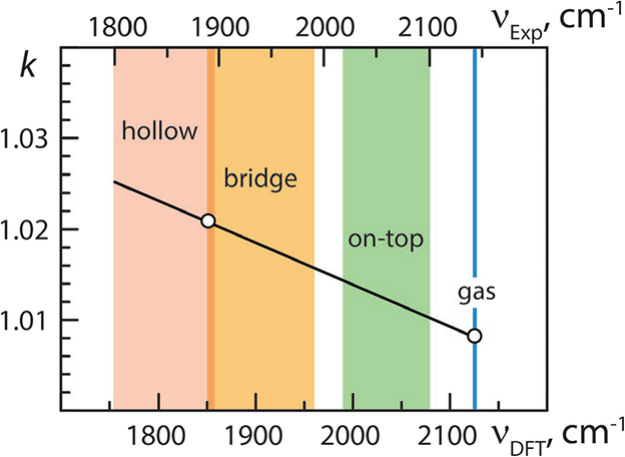
Scaling factor, *k* (solid line, eq 4), relating calculated frequencies of C–O stretching vibrations, ν_DFT_ (cm^–1^), with the corresponding experimental frequencies, ν_Exp_, eq 2. Gas-phase CO and single CO on Pd(001) were used as reference points (open circles) to obtain *k* values. The bands characterizing various modes of CO adsorption (3-fold hollow, bridge, and on-top) on Pd single-crystal surfaces (slabs) and NPs are highlighted. The left and right edges of each band correspond to low (singleton) and high CO coverages, respectively.

The first-principles analysis by Mason et al.^41^ have shown that the correction to the GGA-calculated adsorption energy of CO on transition metal surfaces depends on the coordination of the adsorption site, with the smallest correction introduced for the on-top site, followed by the 2-fold bridge site, the 3-fold site on the (111) surface, and the 4-fold hollow on (100) surfaces. For CO on Pd, the correction Δ *t*o be subtracted from the GGA-calculated adsorption energy is about 20 kJ mol^–1^ for on-top coordination, 40 kJ mol^–1^ for bridge site adsorption, and 55 kJ mol^–1^ for 3-fold hollow.^41^ It was also shown that such site-specific correction linearly correlates with the C–O stretching frequency,^41,42^ which is also site-specific and, in turn, linearly correlates with the C–O distance in the adsorbed species:^43,44^

Δ∼ν(C−O)∼d(C−O) 3

Due to the excess of backdonation increasing with the coordination of adsorbed CO by the adsorption site,^44^ scaling factors higher than that for the gas-phase CO have to be used for correction of the calculated DFT-GGA vibration frequencies for chemisorbed CO. For instance, the scaling factor about 1.021 is used to bring the PBE-calculated C–O stretching frequencies into correspondence with experimental values for bridge-adsorbed CO on Pd(001) single crystals/slabs, leading to almost perfect quantitative agreement between computations and experiment in a wide range of CO coverages.^21,22^ On the other hand, the use at the same computational level of a smaller scaling factor, 1.015,^22^ for the CO/Pd(111) system still leaves space for ambiguity and controversy even in the low-coverage regime because higher backdonation for 3-fold CO adsorption compared to bridge-bonded CO on Pd(001) suggests a stronger excessive frequency shift, and, respectively, a higher scaling factor. Moreover, three adsorption modes with different coordination—on-top, bridge, and 3-fold—have been observed on Pd(111) at different coverages; therefore, the use of a single scaling factor value may not be sufficient for reliable computational modeling of such a complicated system.

For NPs, where various adsorption modes (hollow, bridge, and on-top) may be found at different surface structural elements (regular facets, edges, kinks, and other defects), it is desirable to develop a simple and transparent scaling procedure applicable to all types of adsorption complexes. Based on the correlation ([Disp-formula eq3]) between the site-dependent adsorption energy correction and C–O vibration frequency,^41^ we proposed, in the present study, the frequency scaling factor, *k*, which linearly depends on the C–O stretching frequency:

$$k=1.10657-0.0000463\times \nu_{\mathrm{DFT}}$$ 4

The parametrization of eq 4 is based on the two most reliable experimental references: gas-phase CO (scaling factor 1.0085) and singleton CO on Pd(001) (1.0208). Adsorption of CO on Pd(001) has been well studied both experimentally^16,18^ and by DFT calculations.^21,22^ Only the bridge adsorption mode is observed on Pd(001),^16,18^ corresponding to the lowest-energy adsorption complex in calculations.^21,22^ The linear dependence of the C–O frequency on coverage yields the singleton values (the limit of a single adsorbed molecule) of 1890 and 1851 cm^–1^ in the experiment and PBE computations, respectively, giving the scaling factor of 1.0208 for this case.

The scaling via eq 4 is sufficiently flexible to cover the whole experimental range of C–O stretching vibrations from the gas-phase CO (2143 cm^–1^) to the lowest frequency for 3-fold adsorption on Pd(111) slightly above 1800 cm^–1^ at low coverages. The scaling factor varies in a systematic way from 1.0085 (gas-phase) to 1.025 (3-fold singleton on NPs or low-coverage on Pd(111)), as shown in Figure 3. To verify this scaling procedure, let us consider in more detail the case of CO on Pd(111).

Vibrational frequencies associated with the 3-fold hollow adsorption on Pd(111) at low coverages represent a complicated case because the experimental results of Bradshaw and Hoffman^18^ showed almost constant CO vibrational frequency slightly above 1820 cm^–1^ at the coverages below 0.25 monolayer (ML), unlike the expected linear decrease. However, the recent work by Avanesian et al.^22^ revealed a slow decrease of C–O stretching frequency with coverage decrease, although using fewer measurement points than in the early study.^18^ On the other hand, the DFT data of Avanesian et al. show a nearly perfect linear relationship between coverage and frequency, where lower coverage leads to lower frequency, similar to the trend measured and calculated for CO adsorbed on Pd(001).^22^ The PBE frequencies of 1765 and 1769 cm^–1^, calculated at low CO coverages of 0.06 and 0.08 ML on Pd(111) slabs with 4 × 4 and 3 × 4 unit cells, respectively,^22^ are transformed according to eq 4, giving the scale factor *k* = 1.025 to 1809 and 1813 cm^–1^, which fall in the region below 1820 cm^–1^, where C–O vibrations can be expected at the lowest CO coverage on Pd(111) single crystals, assuming a linear frequency decrease with a coverage decrease. On Pd_201_ and Pd_314_ NPs, we calculated, for single CO molecules adsorbed on 3-fold sites of (111) facets, the vibrational frequencies of 1758 and 1772 cm^–1^, which scale using eq 4 to 1802 and 1815 cm^–1^, respectively, quite close to the scaled low-coverage values for (111) slabs. Thus, the scaling in eq 4 also performs reasonably well in the frequency region, corresponding to the 3-fold adsorption mode at low coverage.

Experimental and theoretical investigations agree that at a high coverage of 0.75 ML on the Pd(111) surface, the adsorbed CO molecules occupy the 3-fold fcc and hcp hollow sites and the atop site to form a (2 × 2)-3CO structure.^18–20^ The published (three-layer slab using the calculated lattice constant for bulk Pd) VASP PBE symmetric vibrational frequencies for the hollow and atop sites on Pd(111) are 1864 and 2075 cm^–1^.^20^ Scaling these frequencies by 1.0203 and 1.0105, according to eq 4, gives 1902 and 2097 cm^–1^, respectively, very close to the experimental values of 1895 and 2100 cm^–1^.^18^ Our calculations, applying a slightly modified computational protocol (six-layer Pd(111) slab with the experimental lattice constant of 389 pm for Pd bulk), resulted in the collective vibrational frequencies of CO molecules adsorbed at the hollow and atop sites of 1871 and 2078 cm^–1^, respectively, which scale upon applying eq 4 *k* = 1.0199; 1.0104 to 1908 and 2100 cm^–1^. Apparently, this scaling procedure provides good numerical accuracy of the calculated CO vibrational frequencies, which is comparable with their uncertainty caused by minor alterations in the computational protocol. Notably, the frequency blue shift of about 100 cm^–1^ for the 3-fold adsorbed CO on Pd(111) due to CO–CO interactions at high coverage versus the low coverage is nearly the same as the shift for the bridge CO on Pd(001). Thus, the quite simple linear scaling procedure of eq 4, relying on just two experimental reference frequencies, is valid to quantitatively describe even such a complicated high-coverage case, where other adsorption modes, on-top and 3-fold, are combined and interact with each other. Thus, after examining a number of available experimentally well-resolved CO/Pd systems, we see no examples where a more elaborate procedure (eventually involving nonlinear terms) is indispensable to get a better correspondence between the C–O frequencies in theory and experiment.

### CO Adsorption Complexes on Pd NPs

Here, based on DFT calculations, we will demonstrate how the bridge-site vibrational mode develops on the edges of Pd NPs as a function of the number of interacting coadsorbed CO molecules and reaches the infinite-size limit. The calculated adsorption energies and vibrational frequencies of the symmetric mode scaled according to the procedure described above (eq 4 and Figure 3) for adsorption complexes of multiple CO molecules on the edges of Pd NPs (Figure 1) and on the infinite edge model (Figure 2) are collected in Table 1 (for more calculated data, see Table S1 of the Supporting Information). The evolution of C–O vibrational frequency (symmetric mode in the case of multiple coadsorbed molecules) is shown in Figure 4.

**Table 1:** Calculated Characteristics of CO Adsorbed on Edges of Pd NPs (Figure 1) and the Periodic Model (Figure 2): Adsorption Energy, *E*_ads_ (kJ mol^–1^), and Frequency of the Symmetric C–O Stretching Vibration, ν_1_ (cm^–1^, Scaled as Described by eq 4)

NP	*n*_CO_/⊖_CO_ ^a^	*E*_ads_, kJ mol^–1^	ν_1_, cm^–1^
Pd_201_	1	–194.4	1886
Pd_264_	1 (central)	–181.8	1890
Pd_314_	1 (terminal)	–187.0	1889
Pd_314_	1 (central)	–188.9	1892
Pd_201_	2	–183.4	1929
Pd_314_	3	–172.8	1949
Pd_264_	4	–170.5	1961
Pd_264_	5	–171.0	1966
Pd_293_	6	–168.0	1971
Periodic Edge			
	0.33^b^	–189.4	1893
	0.5^c^	–186.3	1904
	1.0	–160.1	1989

a *n*_CO_, number of adsorbed CO molecules on a model NP; ⊖_CO_, fraction of the bridge sites occupied by CO on the periodic edge model.

b Slab with unit cell of 102 Pd atoms.

c Slab with unit cell of 68 Pd atoms.

**Figure 4:**
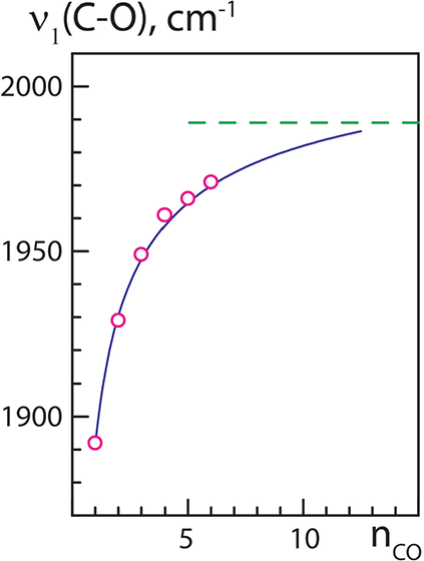
Frequency, ν_1_(*n*), of the fully symmetric C–O stretching vibrational mode as a function of the number of CO molecules, *n*(CO), coadsorbed in the high-density fashion on the edge bridge sites of Pd NPs, as shown in Figure 1. The frequencies for *n*(CO) = 1,..., and 6 are shown by open circles; the dashed line indicates the vibrational frequency calculated for the limiting case of the infinite edge (see Figure 2). The solid line is an approximation by the power function $\nu_{1}(n)=\nu (1)+c\left(1-\frac{1}{n^{\alpha }}\right)$ with the optimized parameters α ≈ 0.5 and *c* = 132 cm^–1^. The frequency values (in cm^–1^) are given after correcting the DFT-calculated values according to eq 4.

### Single CO on the Edges of Pd NPs

First, adsorption of single CO molecules was calculated on edges of Pd_201_, Pd_314_, and Pd_264_ NPs (Table 1). The vibrational frequency on different bridge-edge sites varies from 1886 to 1892 cm^–1^, which is very close to the singleton value for Pd(001), 1890 cm^–1^. In agreement with the concept of generalized coordination numbers,^45,46^ the sites near corners on the short edges of Pd_201_ and Pd_314_ particles adsorb CO slightly stronger than the site in the middle of the long 6Pd edge of Pd_264_. Bridge sites of the shortest 3Pd edge of Pd_201_ exhibit the strongest adsorption, *E*_ads_ = −194 kJ mol^–1^, and, respectively, the lowest calculated vibrational frequency, 1886 cm^–1^.

### CO on the Infinite Edge Model

At the lowest occupation of the periodic edge model, 0.33, where a bridge site occupied by CO is separated from the next one by two vacant sites, the calculated vibrational frequency 1893 cm^–1^ is very close to those of a singleton CO on Pd(001) and isolated CO at edges of Pd_201_, Pd_264_, and Pd_314_ NPs. For a higher occupation, 0.5, the vibrational frequency increases to 1904 cm^–1^, which is still a very low value close to those obtained for Pd(001) at a low CO coverage of 0.125. The calculated adsorption energies for the ridge occupations of 0.33 and 0.5 are also close to each other, −189 and −186 kJ mol^–1^, respectively. Such small changes in the adsorption energy and vibrational frequency with an increase of occupation from 0.33 to 0.5 indicate quite weak CO–CO interactions in this quasi-one-dimensional adsorption system, when the distance between the neighboring CO adsorbates is about 5.6 Å, as for the ridge occupation 0.5. However, for full occupation of the ridge (Figure 2), the adsorption energy substantially decreases in magnitude to −160 kJ mol^–1^ per CO molecule, accompanied by a drastic frequency increase of the symmetric vibrational mode to 1987 cm^–1^ (see the dashed line in Figure 4). This value remarkably well matches the experimentally observed range of 1980–1990 cm^–1^ for CO adsorption on Pd model nanosized catalysts.^6–8^ It is noteworthy that coadsorbed CO molecules at the full edge occupation are very close to each other at about 2.8 Å.

The calculated CO adsorption energy of −160 kJ mol^–1^ on the fully occupied infinite Pd edge is close to −168 kJ mol^–1^ calculated on the Pd(001) surface at a CO coverage of 0.67 ML and notably higher in magnitude than −144 kJ mol^–1^ for a CO coverage of 0.75 ML on the Pd(111) surface,^20^ where one-third of CO adsorbates occupy weakly binding on-top sites. Thus, our calculations advocate the energetic (thermodynamic) feasibility of the full CO occupation of edge bridge sites of Pd NPs well before very high CO coverages on the (001) and (111) facets.

### Ensembles of Multiple CO Molecules on the Edges of Pd NPs

Already for two CO molecules coadsorbed on the neighboring bridge sites at the edge of Pd NPs (Figure 1) a blue shift of the frequency by about 40 cm^–1^ compared to the single adsorbed CO was calculated (Table 1 and Figure 4). For three and four coadsorbed molecules, the frequency further shifts to the higher wavenumbers by 20 and 32 cm^–1^, respectively. Further extension of the CO ensemble to five and six adsorbates leads to a quite moderate frequency increase of about 5 cm^–1^ at each step. For the biggest considered ensemble of six CO coadsorbed molecules, the calculated frequency is about 80 cm^–1^ higher than that of single CO and only about 20 cm^–1^ lower than the value calculated for the periodic model of the infinite edge fully occupied by adsorbed CO. The overall frequency increase from a single CO molecule adsorbed on edges of Pd NPs to the limit of fully occupied by CO infinite edge is about 100 cm^–1^.

We used the following power function to approximate the values of ν_1_(*n*) (Figure 4) corresponding to the number of CO molecules, *n*, ranging from 2 to 6 (the starting value for single CO ν(1) = 1890 cm^–1^):

$$\nu_{1}(n)=\nu (1)+c\left(1-\frac{1}{n^{\alpha }}\right)$$ 5

The fitted values are 132 cm^–1^ for *c* and 0.47 for α, which we set to 0.5 for the sake of simplicity. Extrapolating eq 5 to higher *n* values (solid line in Figure 4) reveals that already for ensembles of about a dozen CO molecules, the symmetric mode frequency ν_1_(*n*) becomes very close to the 1990 cm^–1^ limit calculated for the periodic edge model (dashed line in Figure 4).

As the number of CO molecules in the edge-adsorbed ensemble increases, the average adsorption energy per CO molecule gradually decreases in magnitude to −168.0 kJ mol^–1^ for the largest considered ensemble of six CO molecules on Pd_264_ (Table 1). Unlike the periodic edge model, the finite-size ensembles on edges of Pd NPs exhibit different bindings of CO molecules depending on their positions within the ensemble. As mentioned above, even for a single CO adsorbate, the adsorption energy depends on the adsorption site position on the edge: the adsorption is stronger near the NP corner and weaker in the middle of the edge. However, more important is the repulsive interaction between adsorbed CO molecules within the ensemble. Let us consider the repulsive effect in more detail with the example of the Pd_264_ model. The longest edge of this model consists of six Pd atoms and, hence, can accommodate as many as five bridge-adsorbed CO molecules. The average calculated adsorption energy per CO molecule in such an ensemble of five molecules is −171 kJ mol^–1^ (Table 1), that is about 11 kJ mol^–1^ smaller in magnitude than for a single bridge CO adsorbed in the central site of the 6Pd edge. Evidently, the CO molecule in the middle of this five-member ensemble is the most affected by the repulsive interaction with the coadsorbed CO: the central CO can be desorbed with an energy of 123 kJ mol^–1^, while for the terminal CO of the ensemble, the desorption energy is 173 kJ mol^–1^. Note that the central CO molecule in the five-member ensemble is destabilized notably more than that in the three-member complex Pd_314_(CO)_3_, where the central CO binds to Pd_314_ by 144 kJ mol^–1^.

Here, we should also mention relatively strong adsorption in the two-member ensembles, where two CO molecules occupy the neighboring bridge sites of Pd NP edge (see, for instance, Pd_201_(CO)_2_ in Figure 1) at the distance of only about 280 pm from each other. Two such ensembles separated by a vacant bridge site are formed after desorption of the central CO molecule from the five-member ensemble on Pd_264_ with an adsorption energy of −183 kJ mol^–1^ per CO molecule (essentially the same as for Pd_201_(CO)_2_, see Table 1). This implies a destabilization by only 10 kJ mol^–1^ compared to single adsorbed CO. Evidently, the absence of adsorbed CO adjacent to the two-member ensemble gives the latter sufficient space for relaxation to reduce the quite strong repulsion (cf. the central CO in the five-member ensemble).

Indeed, our models with an occupied edge and bare facets (Figures 1 and 2) are idealized in order to separate the effect of the ensemble size from the influence of total NP coverage by the adsorbate. In real system, the ensembles of multiple CO molecules formed on the edge are expected to interact with CO species adsorbed on the neighboring facet sites, and such interactions may also contribute to the frequency blue shift in ensembles adsorbed on particle edges. The effect of partial occupation of (111) facets was checked for the case of a four-member ensemble on the 5Pd edge of Pd_166_ NP (Figure S3 of the Supporting Information), which is, by its properties, very similar to the four-member ensemble on the 6Pd edge of Pd_264_ shown in Figure 1. The addition of two CO molecules adsorbed on 3-fold hollow sites of (111) facets in the neighborhood of the edge-adsorbed ensemble leads to the increase of the calculated frequency of the symmetric C–O vibrational mode by 3 cm^–1^, that is, noticeably smaller than the effect associated with the increase of the size of the edge-adsorbed ensemble from four to five and six members on longer edges of Pd_264_ and Pd_293_ NPs (Figure 1), where each additional molecule in the ensemble shifts the frequency of the symmetric mode by 5–6 cm^–1^ to higher wavenumbers (Figure 4).

### Mechanistic Aspects of the Vibrational Frequency Shift

It is known that the dipolar interaction is not sufficient to explain the overall frequency shift observed with the increase of CO coverage on metal surfaces.^47^ The mechanism of C–O vibration modes coupling is more complicated, as Loffreda et al. have shown using DFT calculations of CO adsorption on Pd(111), distinguishing the static and dynamic contributions.^19^ To this end, let us consider in more detail the vibrational modes shown in Figure 5 for the ensemble of five CO molecules adsorbed on the edge of Pd_264_.

**Figure 5:**
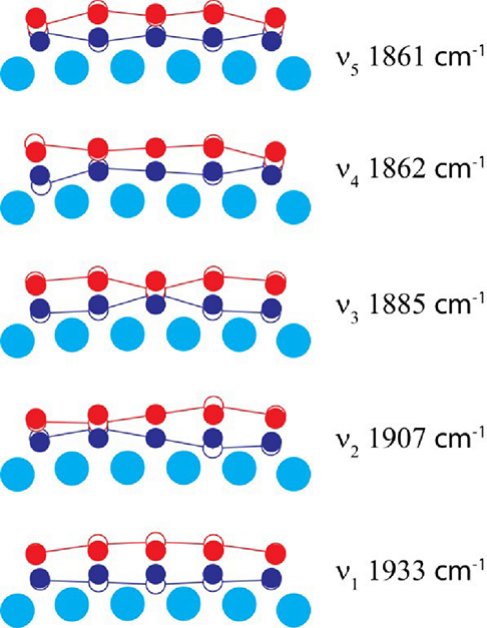
Vibrational modes calculated for five CO molecules adsorbed on the 6Pd edge of the Pd_264_ particle. Solid circles correspond to equilibrium atomic positions, while open circles and solid lines show the displacements for each normal vibration mode. Positions of Pd atoms (light blue) are fixed in calculations, while carbon (dark blue) and oxygen (red) atoms of all CO adsorbates participate in vibrational motions. The not-corrected DFT frequency values are given here.

The static effect arises from the repulsive adsorbate–adsorbate lateral interactions within the ensemble, which weakens the adsorption bonds leading to the reduced backdonation and strengthening of the C–O bond, as manifested by the shorter C–O bond and higher C–O stretching frequency. Hence, the equilibrium bond length and adsorption energy (discussed above) represent the main static characteristics of the molecules in the ensembles.

Being the most affected by the lateral repulsive interaction, the three CO molecules in the middle of the five-member ensemble are very similar to each other by their structural characteristics, exhibiting a C–O bond distance of 117.2 pm (Table S1 of the Supporting Information), while longer bonds of 117.8 pm were calculated for the terminal CO molecules in the ensemble (cf. 118.1 pm for single CO adsorbed on the central site). There is a rather strict linear correlation (see eq S1 of the Supporting Information) between the C–O bond length and the frequency of C–O stretching vibration for single CO adsorbed on NPs, as well as on slabs at low coverage, when the interaction with other adsorbed CO can be neglected. Based on this correlation, the nonscaled DFT frequency about 1920 cm^–1^ is expected for a CO molecule with a bond length of 117.2 pm, isolated from other adsorbed species. However, vibrational motions of each molecule occur in the field of other molecules in the ensemble. The frequency of 1907 cm^–1^ is calculated, when only the central molecule of the ensemble on the 6Pd edge of the Pd_264_ NP vibrated, keeping the nuclei of four other CO molecules frozen at their equilibrium positions. The difference between the latter result and the frequency of single CO adsorbed in the central bridge site at the 6Pd edge of Pd_264_ (1851 cm^–1^, nonscaled) is 56 cm^–1^, which provides an estimate of the static effect.

Simultaneous vibrations of all molecules of the ensemble cause adsorbate–adsorbate vibrational coupling, referred to as the dynamic effect,^19^ see the normal vibrational modes illustrated for the five-member ensemble in Figure 5. The fully symmetric mode, ν_1_, where all members of the ensemble oscillate in the same phase, exhibits the highest frequency, 1933 cm^–1^, and it is the most intense in infrared spectroscopy as a consequence of the largest variation of the electric dipole moment component perpendicular to the surface. Mainly, three CO molecules in the middle of the ensemble (those with similar static properties as characterized by the bond length) contribute to the ν_1_ mode with little admixture from two terminal CO molecules. The dynamic effect adds 26 cm^–1^ to the blue shift, as shown by the difference between ν_1_ and the frequency of 1907 cm^–1^ calculated for the central CO oscillating in the fixed field of the rest of the ensemble.

Notably, the wavenumber 1907 cm^–1^ of the next mode, ν_2_, coincides with the frequency calculated for the central CO oscillating in the fixed environment. This coincidence is not occasional because ν_2_ is produced mainly by two oscillating CO molecules adjacent to fixed central CO (the central CO contribution vanishes here for symmetry reasons) and little contribution from two terminal CO molecules. Thus, roughly, this is also the motion of the CO molecule with quite similar to the central CO static characteristics in the field of fixed neighbors.

The mode ν_3_, 1885 cm^–1^ (close to the CO singleton), is the most complex, with contributions of all molecules of the ensemble. Finally, low-frequency modes ν_4_ and ν_5_ (Figure 5), with the wavenumbers of 1861 and 1862 cm^–1^, respectively, both exhibit dominating contributions from the terminal CO molecules in symmetric and antisymmetric combinations, almost degenerate by energy. Thus, in all modes with the exclusion of ν_3_, vibrational motions of the terminal CO are well separated by energy from those of the CO molecules located inside the ensemble.

Remarkably, the bond lengths of three central CO molecules of Pd_264_(CO)_5_, 117.21 and 117.23 pm, are very close to the bond lengths of the CO molecules at the fully occupied infinite edge, 117.18 pm. Moreover, the C–O bonds, 117.16 pm, of two central molecules of the six-membered ensemble Pd_293_(CO)_6_ essentially reach the infinite limit. This distance comparison indicates that CO molecules in the middle of the five- and six-member ensembles by their static properties are already quite similar to the molecules within the fully occupied infinite model. This finding suggests that the increase in the symmetric mode frequency with increasing numbers of coadsorbed CO molecules beyond five to six ones occurs mostly due to the dynamic effect.

### Role of Particle Size and Morphology

The effect of frequency shift calculated in the present work on Pd NP edges is quite similar by its nature and value to the frequency shift on the Pd(001) surface. In both cases, the vibrational frequency of the bridge mode grows with the increase of CO coverage by 100 cm^–1^ from the singleton, ca. 1890 cm^–1^, up to the high coverage limit ca. 1990 cm^–1^. On Pd(001), the high frequency limit is reached for the two-dimensional adsorbate superstructure at a coverage of about 0.75 ML, while on the edge, our data suggest a linear (quasi-)­one-dimensional chain of CO molecules totally occupying the bridge sites available along the edge. Eventually, both the edges and (001) facets with similar energetic of CO adsorbed at saturation coverage may contribute to the vibrational band around 1990 cm^–1^, which is typically observed for Pd NPs. According to Ouvrard et al., the vibrational band at 1950–1970 cm^–1^ observed on Pd NPs can be explained just by CO adsorption on bridge sites of (001) and (111) facets,^9,10^ while only on-top mode at about 2035–2060 cm^–1^ was assumed to contribute from edge-adsorbed CO molecules. However, our present results strongly suggest that high-coverage CO adsorption at the bridge sites of the Pd edges contributes to the same region of vibrational spectra as that of the high-coverage CO on Pd(001). Evidently, the relation between these two contributions depends on the size and morphology of Pd NPs and a special methodological effort is necessary to distinguish between these contributions in the experiment. Interestingly, neither on facets nor on edges a signal from the bridge mode was detected in experiments performed on the smallest Pd particles.^10^

For well-ordered truncated octahedrons with dominating (111) faceting as Pd_201_ or Pd_314_ (Figure 1, for larger models counting thousands of atoms, see, for instance, modeling of bimetallic nanoalloys^48^), the contribution of (001) facets seems to be of minor importance for statistical reasons, although CO ensembles at high coverage on finite-size (001) facets require a special study in a similar fashion as the present work of CO ensembles on the Pd edges. Evidently, the role of edges in binding adsorbate species increases with the surface-to-volume ratio as the size of NPs decreases. On the other hand, model catalyst particles should be sufficiently big to accommodate an ensemble of coadsorbed CO molecules to exhibit a vibrational band around 1990 cm^–1^. As shown in Figure 4, the correlation eq 5 predicts that the frequency of the symmetric mode for an ensemble of 10–12 CO molecules coadsorbed on the edge is close to the limit of 1990 cm^–1^ calculated for the periodic model. This enables an estimate of the minimum edge length of 3–3.5 nm to accommodate an ensemble of 10–12 coadsorbed CO molecules.

## Conclusions

To strengthen the previous assignment of the experimental CO vibrational spectra on Pd-supported NPs,^15^ we performed DFT modeling of CO adsorption at varying coverages on edges of Pd NPs containing over 200 atoms, with the focus on the frequency of the spectroscopically most intense symmetric C–O stretching vibrational mode. For the accurate description, we proposed a novel frequency-dependent scaling of the DFT C–O frequencies for adsorption on different sites of nanostructured Pd catalysts, enabling quantitative agreement with the reference experimental values. The calculations revealed the energetically stable dense adsorption of multiple CO molecules with the full occupation of the available bridge sites on NP edges. The coupling of vibrating CO molecules with their neighbors in such condensed ensembles leads to a strong frequency increase with the increase in number of CO molecules. Already for two coadsorbed nearby CO molecules, the calculated frequency is blue-shifted from the singleton, 1890 cm^–1^, by about 40 cm^–1^. For the ensemble of six coadsorbed CO molecules, the blue shift increases to 80 cm^–1^, while the overall frequency increase in the limit of fully occupied infinite edge is about 100 cm^–1^. The resulting calculated frequency 1989 cm^–1^ precisely matches the experimental one around 1990 cm^–1^ in Pd/CO nanosystems previously attributed to CO adsorbed on bridge sites on either edges^7,8,15^ or facets (presumably (001)^9,10^ ) of Pd NPs. This frequency is much higher than the singleton frequency of CO on bridge sites of Pd NPs or Pd(001). Hence, the highly blue-shifted vibrational band mainly originates from a collective interaction of coadsorbed CO molecules. This interaction was computationally studied in the literature only for periodic/infinite Pd/CO systems.^19,22^ Now, using the nanosized models with a finite number of coadsorbed CO molecules, we were able to estimate the length of the edge comprising 10–12 Pd atoms (and, hence, the size of NPs, 3–3.5 nm), which, in the case of full occupation by adsorbed CO, can feature the vibrational band at 1990 cm^–1^. Evidently, for smaller particles with shorter edges (and smaller facets), lower frequencies of the vibrational band associated with the bridge-adsorbed CO are expected. The present work on CO adsorption at the edges of Pd NPs is a necessary prerequisite for constructing a complete model description of high-coverage CO adsorption on Pd NPs involving all structural elements, including both regular facets and low-coordinated edges.

## Supplementary Material



## Notes

Structural and energetic data of calculated Pd nanoparticles and their adsorption complexes with CO, along with relevant data for the parent Pd and CO systems (PDF)
