# Supplementary material for: CO Adsorption on Pd Nanoparticles: Assignment of Experimental C–O Vibrational Frequencies by DFT Calculations
Source: J Phys Chem C Nanomater Interfaces. 2026 Feb 5;130(7):2562–70. doi: 10.1021/acs.jpcc.5c08124 (PMC12928204; doi:10.1021/acs.jpcc.5c08124)
Supplement: Supplementary file 1 [file jp5c08124_si_001.pdf]

Supporting Information

CO Adsorption on Pd Nanoparticles: Assignment of Experimental C-O Vibrational Frequencies by DFT Calculations

Ilya V. Yudanov,[†,‡*] *Svetlana S. Laletina*[‡,#] *and Konstantin M. Neyman* [§,||*]

[†] Institute of Solid State Chemistry and Mechanochemistry (ISSCMC) of the Siberian Branch of the Russian Academy of Sciences (SB RAS), 630128 Novosibirsk, Russia;
[‡] Boreskov Institute of Catalysis (BIC), SB RAS, 630090 Novosibirsk, Russia;
[#] Institute of Chemistry and Chemical Technology (ICCT), SB RAS, 660036 Krasnoyarsk, Russia
[§] Departament de Ciència de Materials i Química Física and Institut de Quimica Teòrica i Computacional, Universitat de Barcelona, c/Martí i Franquès 1, 08028 Barcelona, Spain

[||] ICREA (Institució Catalana de Recerca i Estudis Avancats), Pg. Lluís Companys 23, 08010 Barcelona, Spain

CORRESPONDING AUTHOR FOOTNOTE:
* (I.V.Y.) E-mail: yudanov@catalysis.ru.
* (K.M.N.) E-mail: konstantin.neyman@icrea.cat.

Content of Suppoting Information:

Table S1. Calculated characteristics of CO adsorbed on the edges of Pd NPs and on the periodic slab models.

Figure S1. CO adsorption on Pd(001) as a reference for scaling the DFT-calculated frequencies.

Table S2. Characteristics of CO adsorption on Pd(001) surface for different coverages.

Figure S2. Views of condensed CO phase on Pd(001) at θ_{CO} = 0.6667 ML.

Equation S1. Correlation between C-O bond length and the frequency of C-O vibration.

Figure S3. Adsorption complex $Pd_{166}(CO)_6$.

Table S3. Calculated by DFT atomic coordinates and total energies of key structures discussed in the article.

Table S1. Calculated characteristics of CO adsorbed on the edges of Pd NPs (Fig. 1 of the main manuscript) and on the periodic slab models (Fig. 2).

NP	n_{CO}	E_{ads}, eV	d(C-O), pm	ν_{DFT}, cm^{-1}	Scale factor	ν_{scaled}, cm^{-1}
Pd_{201}	1	-2.015	118.2	1847.6	1.0210	1886
Pd_{264}	1 [a]	-1.884	118.10	1851.2	1.0208	1890
Pd_{314}	1 [a]	-1.958	118.12	1853.4	1.0208	1892
Pd_{314}	1 [b]	-1.938	118.08	1850.8	1.0209	1889
Pd_{201}	2	-1.901	117.67×2	1893.4<br>1865.1	1.0189	1929 [c]
Pd_{314}	3	-1.791	117.31<br>117.64×2	1915.2<br>1875.8<br>1863.9	1.0179	1949 [c]
Pd_{264}	4	-1.767	117.23<br>117.24<br>117.65<br>117.77	1927.5<br>1895.7<br>1868.7<br>1860.5	1.0173	1961 [c]
Pd_{264}	5	-1.772	117.21<br>117.23×2<br>117.75×2	1932.7<br>1907.6<br>1884.9<br>1862.4<br>1861.1	1.0170	1966 [c]
Pd_{293}	6	-1.741	117.16×2<br>117.28×2<br>117.65×2	1938.4<br>1916.5<br>1897.5<br>1882.7<br>1865.8<br>1864.9	1.0168	1971 [c]
Pd slab with infinite edge	1.0	-1.648	117.16×2	1957.6<br>1878.6	1.0159	1989 [c]
	0.5	-1.931	117.93	1867.0	1.0201	1905 [c]
	0.33	-1.963	118.08	1854.4	1.0207	1893 [c]

[a] in the central bridge position of the edge;

[b] in the terminal bridge position of the edge;

[c] symmetric collective vibrational frequency of all adsorbed CO molecules.

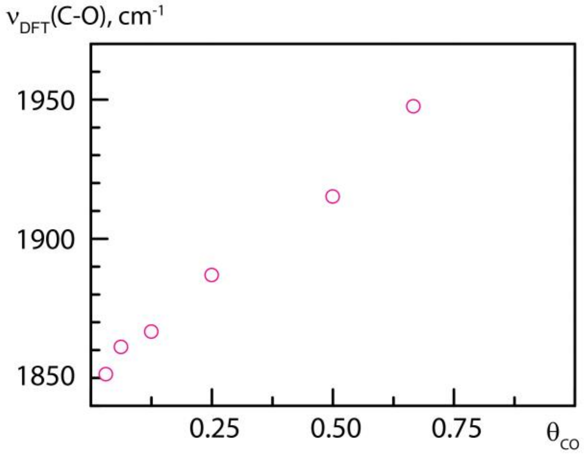

Figure S1. Calculated unscaled frequency of C-O stretching vibration, ν_{DFT}, on Pd(001) surface as a function of CO coverage, θ_{CO}. The linear fit for coverages up to θ(CO)=0.5 yields 1850.6 cm^{-1} as a value zero coverage limit. Thus, the singleton frequency is estimated at 1851 cm^{-1}. Scaling factor 1.0208 is used to establish the correspondence between theoretical and observed singleton frequencies, 1851 and 1890 cm^{-1}, respectively.

Table S2. Characteristics of CO adsorption on Pd(001) surface for different coverages.

Pd(001) slab	θ_{CO}	E_{ads}, eV	d(C-O), pm	ν_{DFT}, cm^{-1}	Scale factor, k [a]	$\nu_{DFT}\times k$, cm^{-1}	ν_{Exp} cm^{-1}
Singleton				1851	1.0208	1889	1890
CO, Pd_{160}	0.03125	-1.919	118.09	1851.3	1.0208	1890	
CO, Pd_{80}	0.0625	-1.915	118.00	1861.1	1.0203	1899	
CO, Pd_{40}	0.125	-1.904	117.96	1866.6	1.0201	1904	
CO, Pd_{20}	0.25	-1.940	117.81	1886.9	1.0191	1923	
2CO, Pd_{20}	0.5	-1.924	117.58	1915.1	1.0178	1949	1949.5
4CO, Pd_{30}	0.66667	-1.741	117.29	1947.5	1.0163	1979	1973

In the case of multiple CO molecules in the unit cell the vibrational frequency is given only for the symmetric mode. Scaling factor, *k*, linearly depends on the calculated frequency according to Eq (4) of the main manuscript. Experimantal values, ν_{Exp}, according to ref. 21 of the main manuscript. <Ouvrard, A.; Wang, J. J.; Ghalgaoui, A.; Nave, S.; Carrez, S.; Zheng, W. Q.; Dubost, H.; Bourguignon, B. CO Adsorption on Pd(100) Revisited by Sum Frequency Generation: Evidence for Two Adsorption Sites in the Compression Stage. *J. Phys. Chem. C* **2014**, *118*, 19688−19700.>

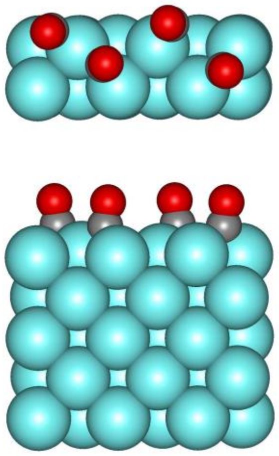

Figure S2. CO adsorption on Pd(001): top and side view of condensed phase at θ_{CO} = 0.6667 ML. The unit cell contains four CO molecules and 30 Pd atoms (five-layer slab). This structure is comparable to the fully occupied infinite edge model (Fig. 2 of the main manuscript) by the blue shift of C-O vibartional frequnce

Equation S1. Correlation between C-O bond length and the frequency of C-O vibration.

$$\nu_{DFT} = 10341 - 7185 * d_{CO}$$

The dependence of vibrational frequency (cm^{-1}) on the bond length in CO molecule, d_{CO} (pm), calculated for single CO on various sites of Pd NPs, CO on Pd(001) and Pd(111) at low coverage and gas-phase CO. The the correlation coefficient is 0.9995.

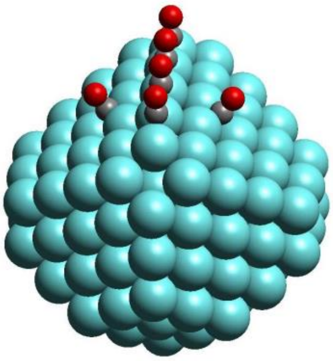

Figure S3. Adsorption complex $Pd_{166}(CO)_6$, where four CO molecules are adsorbed at the bridge sites of 5Pd edge and two CO molecules at three-fold hollow sites of the neighboring (111) facets of Pd_{166} particle. The frequency of the symmetric mode is calculated at 1929.4 cm^{-1} (PBE without correction) that is by 3 cm^{-1} higher than for an ensemble of four CO on the edge in the absence of adsorbate on (111) facets ($Pd_{166}(CO)_4$, 1926.3cm^{-1}).

Table S3. Calculated by DFT atomic coordinates and total energies of key structures discussed in the article.

NP Pd_{201}

-938.457 eV

Pd	12.50000000000000	12.50000000000000	12.50000000000000
Pd	10.52229604263261	12.50000000000000	10.52229604263261
Pd	12.50000000000000	10.52229604263261	10.52229604263261
Pd	10.52229604263261	10.52229604263261	12.50000000000000
Pd	10.52229604263261	12.50000000000000	14.47770395736739
Pd	14.47770395736739	12.50000000000000	10.52229604263261
Pd	12.50000000000000	14.47770395736739	10.52229604263261
Pd	12.50000000000000	10.52229604263261	14.47770395736739
Pd	14.47770395736739	10.52229604263261	12.50000000000000
Pd	10.52229604263261	14.47770395736739	12.50000000000000
Pd	14.47770395736739	12.50000000000000	14.47770395736739
Pd	12.50000000000000	14.47770395736739	14.47770395736739
Pd	14.47770395736739	14.47770395736739	12.50000000000000
Pd	12.50000000000000	16.40026262542458	12.50000000000000
Pd	16.40026262542458	12.50000000000000	12.50000000000000
Pd	12.50000000000000	12.50000000000000	16.40026262542458
Pd	12.50000000000000	8.59973737457542	12.50000000000000
Pd	8.59973737457542	12.50000000000000	12.50000000000000
Pd	12.50000000000000	12.50000000000000	8.59973737457542
Pd	16.42883014756374	14.50048948653452	10.49951051346548
Pd	16.42883014756374	10.49951051346548	14.50048948653452
Pd	10.49951051346548	16.42883014756374	14.50048948653452
Pd	14.50048948653452	16.42883014756374	10.49951051346548
Pd	14.50048948653452	10.49951051346548	16.42883014756374
Pd	10.49951051346548	14.50048948653452	16.42883014756374
Pd	14.50048948653452	16.42883014756374	14.50048948653452
Pd	10.49951051346548	16.42883014756374	10.49951051346548
Pd	16.42883014756374	10.49951051346548	10.49951051346548
Pd	16.42883014756374	14.50048948653452	14.50048948653452
Pd	10.49951051346548	10.49951051346548	16.42883014756374
Pd	14.50048948653452	14.50048948653452	16.42883014756374
Pd	8.57116985243626	10.49951051346548	10.49951051346548
Pd	8.57116985243626	14.50048948653452	14.50048948653452
Pd	10.49951051346548	8.57116985243626	10.49951051346548
Pd	14.50048948653452	8.57116985243626	14.50048948653452
Pd	10.49951051346548	10.49951051346548	8.57116985243626
Pd	14.50048948653452	14.50048948653452	8.57116985243626
Pd	10.49951051346548	8.57116985243626	14.50048948653452
Pd	14.50048948653452	8.57116985243626	10.49951051346548
Pd	8.57116985243626	10.49951051346548	14.50048948653452
Pd	8.57116985243626	14.50048948653452	10.49951051346548
Pd	10.49951051346548	14.50048948653452	8.57116985243626
Pd	14.50048948653452	10.49951051346548	8.57116985243626
Pd	8.56989903347806	8.56989903347806	12.50000000000000
Pd	16.43010096652194	16.43010096652194	12.50000000000000
Pd	16.43010096652194	8.56989903347806	12.50000000000000
Pd	8.56989903347806	16.43010096652194	12.50000000000000
Pd	12.50000000000000	8.56989903347806	8.56989903347806
Pd	12.50000000000000	16.43010096652194	16.43010096652194
Pd	12.50000000000000	16.43010096652194	8.56989903347806
Pd	12.50000000000000	8.56989903347806	16.43010096652194
Pd	8.56989903347806	12.50000000000000	8.56989903347806
Pd	16.43010096652194	12.50000000000000	16.43010096652194
Pd	8.56989903347806	12.50000000000000	16.43010096652194

Pd	16.43010096652194	12.50000000000000	8.56989903347806
Pd	14.46608561187854	12.50000000000000	6.71687998282495
Pd	14.46608561187854	12.50000000000000	18.28312001717505
Pd	6.71687998282495	12.50000000000000	14.46608561187854
Pd	18.28312001717505	12.50000000000000	14.46608561187854
Pd	12.50000000000000	6.71687998282495	14.46608561187854
Pd	12.50000000000000	18.28312001717505	14.46608561187854
Pd	6.71687998282495	14.46608561187854	12.50000000000000
Pd	18.28312001717505	14.46608561187854	12.50000000000000
Pd	12.50000000000000	14.46608561187854	18.28312001717505
Pd	12.50000000000000	14.46608561187854	6.71687998282495
Pd	14.46608561187854	6.71687998282495	12.50000000000000
Pd	14.46608561187854	18.28312001717505	12.50000000000000
Pd	10.53391438812146	12.50000000000000	6.71687998282495
Pd	10.53391438812146	12.50000000000000	18.28312001717505
Pd	6.71687998282495	12.50000000000000	10.53391438812146
Pd	18.28312001717505	12.50000000000000	10.53391438812146
Pd	12.50000000000000	6.71687998282495	10.53391438812146
Pd	12.50000000000000	18.28312001717505	10.53391438812146
Pd	6.71687998282495	10.53391438812146	12.50000000000000
Pd	18.28312001717505	10.53391438812146	12.50000000000000
Pd	12.50000000000000	10.53391438812146	18.28312001717505
Pd	12.50000000000000	10.53391438812146	6.71687998282495
Pd	10.53391438812146	6.71687998282495	12.50000000000000
Pd	10.53391438812146	18.28312001717505	12.50000000000000
Pd	16.50002733726306	16.50002733726306	16.50002733726306
Pd	16.50002733726306	16.50002733726306	8.49997266273694
Pd	16.50002733726306	8.49997266273694	16.50002733726306
Pd	8.49997266273694	16.50002733726306	16.50002733726306
Pd	8.49997266273694	8.49997266273694	16.50002733726306
Pd	8.49997266273694	16.50002733726306	8.49997266273694
Pd	16.50002733726306	8.49997266273694	8.49997266273694
Pd	8.49997266273694	8.49997266273694	8.49997266273694
Pd	18.39898107273513	16.47414036180625	14.53886041930568
Pd	16.47414036180625	14.53886041930568	18.39898107273513
Pd	14.53886041930568	18.39898107273513	16.47414036180625
Pd	16.47414036180625	18.39898107273513	14.53886041930568
Pd	14.53886041930568	16.47414036180625	18.39898107273513
Pd	18.39898107273513	14.53886041930568	16.47414036180625
Pd	16.47414036180625	6.60101892726487	14.53886041930568
Pd	6.60101892726487	14.53886041930568	16.47414036180625
Pd	6.60101892726487	16.47414036180625	14.53886041930568
Pd	14.53886041930568	6.60101892726487	16.47414036180625
Pd	14.53886041930568	16.47414036180625	6.60101892726487
Pd	16.47414036180625	14.53886041930568	6.60101892726487
Pd	16.47414036180625	18.39898107273513	10.46113958069432
Pd	18.39898107273513	10.46113958069432	16.47414036180625
Pd	18.39898107273513	16.47414036180625	10.46113958069432
Pd	10.46113958069432	18.39898107273513	16.47414036180625
Pd	10.46113958069432	16.47414036180625	18.39898107273513
Pd	16.47414036180625	10.46113958069432	18.39898107273513
Pd	6.60101892726487	16.47414036180625	10.46113958069432
Pd	16.47414036180625	10.46113958069432	6.60101892726487
Pd	10.46113958069432	6.60101892726487	16.47414036180625
Pd	16.47414036180625	6.60101892726487	10.46113958069432
Pd	10.46113958069432	16.47414036180625	6.60101892726487
Pd	6.60101892726487	10.46113958069432	16.47414036180625
Pd	8.52585963819375	18.39898107273513	14.53886041930568
Pd	14.53886041930568	8.52585963819375	18.39898107273513
Pd	18.39898107273513	14.53886041930568	8.52585963819375

Pd	18.39898107273513	8.52585963819375	14.53886041930568
Pd	8.52585963819375	14.53886041930568	18.39898107273513
Pd	14.53886041930568	18.39898107273513	8.52585963819375
Pd	6.60101892726487	8.52585963819375	14.53886041930568
Pd	8.52585963819375	14.53886041930568	6.60101892726487
Pd	14.53886041930568	6.60101892726487	8.52585963819375
Pd	8.52585963819375	6.60101892726487	14.53886041930568
Pd	14.53886041930568	8.52585963819375	6.60101892726487
Pd	6.60101892726487	14.53886041930568	8.52585963819375
Pd	18.39898107273513	8.52585963819375	10.46113958069432
Pd	8.52585963819375	10.46113958069432	18.39898107273513
Pd	10.46113958069432	18.39898107273513	8.52585963819375
Pd	8.52585963819375	18.39898107273513	10.46113958069432
Pd	10.46113958069432	8.52585963819375	18.39898107273513
Pd	18.39898107273513	10.46113958069432	8.52585963819375
Pd	8.52585963819375	6.60101892726487	10.46113958069432
Pd	10.46113958069432	8.52585963819375	6.60101892726487
Pd	6.60101892726487	10.46113958069432	8.52585963819375
Pd	6.60101892726487	8.52585963819375	10.46113958069432
Pd	8.52585963819375	10.46113958069432	6.60101892726487
Pd	10.46113958069432	6.60101892726487	8.52585963819375
Pd	4.72081899598969	12.50000000000000	12.50000000000000
Pd	12.50000000000000	4.72081899598969	12.50000000000000
Pd	12.50000000000000	12.50000000000000	4.72081899598969
Pd	20.27918100401031	12.50000000000000	12.50000000000000
Pd	12.50000000000000	20.27918100401031	12.50000000000000
Pd	12.50000000000000	12.50000000000000	20.27918100401031
Pd	20.24834500059281	14.42967462803956	10.57032537196045
Pd	20.24834500059281	10.57032537196045	14.42967462803956
Pd	10.57032537196045	20.24834500059281	14.42967462803956
Pd	14.42967462803956	20.24834500059281	10.57032537196045
Pd	14.42967462803956	10.57032537196045	20.24834500059281
Pd	10.57032537196045	14.42967462803956	20.24834500059281
Pd	14.42967462803956	20.24834500059281	14.42967462803956
Pd	10.57032537196045	20.24834500059281	10.57032537196045
Pd	20.24834500059281	10.57032537196045	10.57032537196045
Pd	20.24834500059281	14.42967462803956	14.42967462803956
Pd	10.57032537196045	10.57032537196045	20.24834500059281
Pd	14.42967462803956	14.42967462803956	20.24834500059281
Pd	4.75165499940719	10.57032537196045	10.57032537196045
Pd	4.75165499940719	14.42967462803956	14.42967462803956
Pd	10.57032537196045	4.75165499940719	10.57032537196045
Pd	14.42967462803956	4.75165499940719	14.42967462803956
Pd	10.57032537196045	10.57032537196045	4.75165499940719
Pd	14.42967462803956	14.42967462803956	4.75165499940719
Pd	10.57032537196045	4.75165499940719	14.42967462803956
Pd	14.42967462803956	4.75165499940719	10.57032537196045
Pd	4.75165499940719	10.57032537196045	14.42967462803956
Pd	4.75165499940719	14.42967462803956	10.57032537196045
Pd	10.57032537196045	14.42967462803956	4.75165499940719
Pd	14.42967462803956	10.57032537196045	4.75165499940719
Pd	12.50000000000000	6.66987094172809	6.66987094172809
Pd	6.66987094172809	12.50000000000000	6.66987094172809
Pd	6.66987094172809	6.66987094172809	12.50000000000000
Pd	12.50000000000000	18.33012905827191	6.66987094172809
Pd	12.50000000000000	6.66987094172809	18.33012905827191
Pd	12.50000000000000	18.33012905827191	18.33012905827191
Pd	6.66987094172809	12.50000000000000	18.33012905827191
Pd	18.33012905827191	12.50000000000000	6.66987094172809
Pd	18.33012905827191	12.50000000000000	18.33012905827191

```
Pd   18.33012905827191   6.66987094172809   12.50000000000000
Pd   6.66987094172809   18.33012905827191   12.50000000000000
Pd   18.33012905827191   18.33012905827191   12.50000000000000
Pd   4.79232892487840   8.66591943026658   12.50000000000000
Pd   12.50000000000000   4.79232892487840   8.66591943026658
Pd   8.66591943026658   12.50000000000000   4.79232892487840
Pd   8.66591943026658   4.79232892487840   12.50000000000000
```

NP Pd_{314}

```
-1490.200 eV
Pd   1.972329129   0   0
Pd   -1.972329129   0   0
Pd   0   1.972329129   0
Pd   0   -1.972329129   0
Pd   0   0   1.972329129
Pd   0   0   -1.972329129
Pd   1.978420994   1.978420994   1.978420994
Pd   1.978420994   -1.978420994   -1.978420994
Pd   -1.978420994   1.978420994   -1.978420994
Pd   -1.978420994   -1.978420994   1.978420994
Pd   1.978420994   -1.978420994   1.978420994
Pd   1.978420994   1.978420994   -1.978420994
Pd   -1.978420994   1.978420994   1.978420994
Pd   -1.978420994   -1.978420994   -1.978420994
Pd   3.9303222   1.990960558   0
Pd   3.9303222   -1.990960558   0
Pd   -3.9303222   1.990960558   0
Pd   -3.9303222   -1.990960558   0
Pd   0   3.9303222   1.990960558
Pd   0   3.9303222   -1.990960558
Pd   0   -3.9303222   1.990960558
Pd   0   -3.9303222   -1.990960558
Pd   1.990960558   0   3.9303222
Pd   1.990960558   0   -3.9303222
Pd   -1.990960558   0   -3.9303222
Pd   -1.990960558   0   3.9303222
Pd   3.9303222   0   1.990960558
Pd   0   1.990960558   -3.9303222
Pd   -1.990960558   3.9303222   0
Pd   3.9303222   0   -1.990960558
Pd   0   1.990960558   3.9303222
Pd   1.990960558   -3.9303222   0
Pd   1.990960558   3.9303222   0
Pd   -1.990960558   -3.9303222   0
Pd   0   -1.990960558   3.9303222
Pd   -3.9303222   0   1.990960558
Pd   0   -1.990960558   -3.9303222
Pd   -3.9303222   0   -1.990960558
Pd   5.83054834   0   0
Pd   -5.83054834   0   0
Pd   0   5.83054834   0
Pd   0   -5.83054834   0
Pd   0   0   5.83054834
Pd   0   0   -5.83054834
Pd   3.956964694   3.956964694   2.015440286
Pd   3.956964694   -3.956964694   -2.015440286
Pd   -3.956964694   3.956964694   -2.015440286
Pd   -3.956964694   -3.956964694   2.015440286
Pd   2.015440286   3.956964694   3.956964694
```

Pd	-2.015440286	3.956964694	-3.956964694
Pd	-2.015440286	-3.956964694	3.956964694
Pd	2.015440286	-3.956964694	-3.956964694
Pd	3.956964694	2.015440286	3.956964694
Pd	3.956964694	-2.015440286	-3.956964694
Pd	-3.956964694	2.015440286	-3.956964694
Pd	-3.956964694	-2.015440286	3.956964694
Pd	3.956964694	-2.015440286	3.956964694
Pd	2.015440286	3.956964694	-3.956964694
Pd	-3.956964694	3.956964694	2.015440286
Pd	3.956964694	2.015440286	-3.956964694
Pd	-2.015440286	3.956964694	3.956964694
Pd	3.956964694	-3.956964694	2.015440286
Pd	3.956964694	3.956964694	-2.015440286
Pd	-3.956964694	-3.956964694	-2.015440286
Pd	2.015440286	-3.956964694	3.956964694
Pd	-3.956964694	2.015440286	3.956964694
Pd	-2.015440286	-3.956964694	-3.956964694
Pd	-3.956964694	-2.015440286	-3.956964694
Pd	5.852500934	2.004162678	2.004162678
Pd	5.852500934	-2.004162678	-2.004162678
Pd	-5.852500934	2.004162678	-2.004162678
Pd	-5.852500934	-2.004162678	2.004162678
Pd	2.004162678	5.852500934	2.004162678
Pd	-2.004162678	5.852500934	-2.004162678
Pd	-2.004162678	-5.852500934	2.004162678
Pd	2.004162678	-5.852500934	-2.004162678
Pd	2.004162678	2.004162678	5.852500934
Pd	2.004162678	-2.004162678	-5.852500934
Pd	-2.004162678	2.004162678	-5.852500934
Pd	-2.004162678	-2.004162678	5.852500934
Pd	5.852500934	-2.004162678	2.004162678
Pd	2.004162678	2.004162678	-5.852500934
Pd	-2.004162678	5.852500934	2.004162678
Pd	5.852500934	2.004162678	-2.004162678
Pd	-2.004162678	2.004162678	5.852500934
Pd	2.004162678	-5.852500934	2.004162678
Pd	2.004162678	5.852500934	-2.004162678
Pd	-2.004162678	-5.852500934	-2.004162678
Pd	2.004162678	-2.004162678	5.852500934
Pd	-5.852500934	2.004162678	2.004162678
Pd	-2.004162678	-2.004162678	-5.852500934
Pd	-5.852500934	-2.004162678	-2.004162678
Pd	5.851861652	3.953183835	0
Pd	5.851861652	-3.953183835	0
Pd	-5.851861652	3.953183835	0
Pd	-5.851861652	-3.953183835	0
Pd	0	5.851861652	3.953183835
Pd	0	5.851861652	-3.953183835
Pd	0	-5.851861652	3.953183835
Pd	0	-5.851861652	-3.953183835
Pd	3.953183835	0	5.851861652
Pd	3.953183835	0	-5.851861652
Pd	-3.953183835	0	-5.851861652
Pd	-3.953183835	0	5.851861652
Pd	5.851861652	0	3.953183835
Pd	0	3.953183835	-5.851861652
Pd	-3.953183835	5.851861652	0
Pd	5.851861652	0	-3.953183835
Pd	0	3.953183835	5.851861652

Pd	3.953183835	-5.851861652	0
Pd	3.953183835	5.851861652	0
Pd	-3.953183835	-5.851861652	0
Pd	0	-3.953183835	5.851861652
Pd	-5.851861652	0	3.953183835
Pd	0	-3.953183835	-5.851861652
Pd	-5.851861652	0	-3.953183835
Pd	7.705084582	1.970391599	0
Pd	7.705084582	-1.970391599	0
Pd	-7.705084582	1.970391599	0
Pd	-7.705084582	-1.970391599	0
Pd	0	7.705084582	1.970391599
Pd	0	7.705084582	-1.970391599
Pd	0	-7.705084582	1.970391599
Pd	0	-7.705084582	-1.970391599
Pd	1.970391599	0	7.705084582
Pd	1.970391599	0	-7.705084582
Pd	-1.970391599	0	-7.705084582
Pd	-1.970391599	0	7.705084582
Pd	7.705084582	0	1.970391599
Pd	0	1.970391599	-7.705084582
Pd	-1.970391599	7.705084582	0
Pd	7.705084582	0	-1.970391599
Pd	0	1.970391599	7.705084582
Pd	1.970391599	-7.705084582	0
Pd	1.970391599	7.705084582	0
Pd	-1.970391599	-7.705084582	0
Pd	0	-1.970391599	7.705084582
Pd	-7.705084582	0	1.970391599
Pd	0	-1.970391599	-7.705084582
Pd	-7.705084582	0	-1.970391599
Pd	5.953106299	4.019971238	4.019971238
Pd	5.953106299	-4.019971238	-4.019971238
Pd	-5.953106299	4.019971238	-4.019971238
Pd	-5.953106299	-4.019971238	4.019971238
Pd	4.019971238	5.953106299	4.019971238
Pd	-4.019971238	5.953106299	-4.019971238
Pd	-4.019971238	-5.953106299	4.019971238
Pd	4.019971238	-5.953106299	-4.019971238
Pd	4.019971238	4.019971238	5.953106299
Pd	4.019971238	-4.019971238	-5.953106299
Pd	-4.019971238	4.019971238	-5.953106299
Pd	-4.019971238	-4.019971238	5.953106299
Pd	5.953106299	-4.019971238	4.019971238
Pd	4.019971238	4.019971238	-5.953106299
Pd	-4.019971238	5.953106299	4.019971238
Pd	5.953106299	4.019971238	-4.019971238
Pd	-4.019971238	4.019971238	5.953106299
Pd	4.019971238	-5.953106299	4.019971238
Pd	4.019971238	5.953106299	-4.019971238
Pd	-4.019971238	-5.953106299	-4.019971238
Pd	4.019971238	-4.019971238	5.953106299
Pd	-5.953106299	4.019971238	4.019971238
Pd	-4.019971238	-4.019971238	-5.953106299
Pd	-5.953106299	-4.019971238	-4.019971238
Pd	5.929344298	5.929344298	2.071891945
Pd	5.929344298	-5.929344298	-2.071891945
Pd	-5.929344298	5.929344298	-2.071891945
Pd	-5.929344298	-5.929344298	2.071891945
Pd	2.071891945	5.929344298	5.929344298

Pd	-2.071891945	5.929344298	-5.929344298
Pd	-2.071891945	-5.929344298	5.929344298
Pd	2.071891945	-5.929344298	-5.929344298
Pd	5.929344298	2.071891945	5.929344298
Pd	5.929344298	-2.071891945	-5.929344298
Pd	-5.929344298	2.071891945	-5.929344298
Pd	-5.929344298	-2.071891945	5.929344298
Pd	5.929344298	-2.071891945	5.929344298
Pd	2.071891945	5.929344298	-5.929344298
Pd	-5.929344298	5.929344298	2.071891945
Pd	5.929344298	2.071891945	-5.929344298
Pd	-2.071891945	5.929344298	5.929344298
Pd	5.929344298	-5.929344298	2.071891945
Pd	5.929344298	5.929344298	-2.071891945
Pd	-5.929344298	-5.929344298	-2.071891945
Pd	2.071891945	-5.929344298	5.929344298
Pd	-5.929344298	2.071891945	5.929344298
Pd	-2.071891945	-5.929344298	-5.929344298
Pd	-5.929344298	-2.071891945	-5.929344298
Pd	7.817541201	3.973970835	2.034900045
Pd	7.817541201	-3.973970835	-2.034900045
Pd	-7.817541201	3.973970835	-2.034900045
Pd	-7.817541201	-3.973970835	2.034900045
Pd	2.034900045	7.817541201	3.973970835
Pd	-2.034900045	7.817541201	-3.973970835
Pd	-2.034900045	-7.817541201	3.973970835
Pd	2.034900045	-7.817541201	-3.973970835
Pd	3.973970835	2.034900045	7.817541201
Pd	3.973970835	-2.034900045	-7.817541201
Pd	-3.973970835	2.034900045	-7.817541201
Pd	-3.973970835	-2.034900045	7.817541201
Pd	7.817541201	-2.034900045	3.973970835
Pd	2.034900045	3.973970835	-7.817541201
Pd	-3.973970835	7.817541201	2.034900045
Pd	7.817541201	2.034900045	-3.973970835
Pd	-2.034900045	3.973970835	7.817541201
Pd	3.973970835	-7.817541201	2.034900045
Pd	3.973970835	7.817541201	-2.034900045
Pd	-3.973970835	-7.817541201	-2.034900045
Pd	2.034900045	-3.973970835	7.817541201
Pd	-7.817541201	2.034900045	3.973970835
Pd	-2.034900045	-3.973970835	-7.817541201
Pd	-7.817541201	-2.034900045	-3.973970835
Pd	-7.817541201	-3.973970835	-2.034900045
Pd	-7.817541201	3.973970835	2.034900045
Pd	7.817541201	-3.973970835	2.034900045
Pd	7.817541201	3.973970835	-2.034900045
Pd	-2.034900045	-7.817541201	-3.973970835
Pd	2.034900045	-7.817541201	3.973970835
Pd	2.034900045	7.817541201	-3.973970835
Pd	-2.034900045	7.817541201	3.973970835
Pd	-3.973970835	-2.034900045	-7.817541201
Pd	-3.973970835	2.034900045	7.817541201
Pd	3.973970835	-2.034900045	7.817541201
Pd	3.973970835	2.034900045	-7.817541201
Pd	-7.817541201	2.034900045	-3.973970835
Pd	-2.034900045	-3.973970835	7.817541201
Pd	3.973970835	-7.817541201	-2.034900045
Pd	-7.817541201	-2.034900045	3.973970835
Pd	2.034900045	-3.973970835	-7.817541201

Pd	-3.973970835	7.817541201	-2.034900045
Pd	-3.973970835	-7.817541201	2.034900045
Pd	3.973970835	7.817541201	2.034900045
Pd	-2.034900045	3.973970835	-7.817541201
Pd	7.817541201	-2.034900045	-3.973970835
Pd	2.034900045	3.973970835	7.817541201
Pd	7.817541201	2.034900045	3.973970835
Pd	7.748058379	0	5.843300875
Pd	7.748058379	0	-5.843300875
Pd	-7.748058379	0	-5.843300875
Pd	-7.748058379	0	5.843300875
Pd	5.843300875	7.748058379	0
Pd	-5.843300875	7.748058379	0
Pd	-5.843300875	-7.748058379	0
Pd	5.843300875	-7.748058379	0
Pd	0	5.843300875	7.748058379
Pd	0	-5.843300875	-7.748058379
Pd	0	5.843300875	-7.748058379
Pd	0	-5.843300875	7.748058379
Pd	7.748058379	-5.843300875	0
Pd	5.843300875	0	-7.748058379
Pd	0	7.748058379	5.843300875
Pd	7.748058379	5.843300875	0
Pd	-5.843300875	0	7.748058379
Pd	0	-7.748058379	5.843300875
Pd	0	7.748058379	-5.843300875
Pd	0	-7.748058379	-5.843300875
Pd	5.843300875	0	7.748058379
Pd	-7.748058379	5.843300875	0
Pd	-5.843300875	0	-7.748058379
Pd	-7.748058379	-5.843300875	0
Pd	9.697530227	0	0
Pd	-9.697530227	0	0
Pd	0	9.697530227	0
Pd	0	-9.697530227	0
Pd	0	0	9.697530227
Pd	0	0	-9.697530227
Pd	9.67290319	1.92345098	1.92345098
Pd	9.67290319	-1.92345098	-1.92345098
Pd	-9.67290319	1.92345098	-1.92345098
Pd	-9.67290319	-1.92345098	1.92345098
Pd	1.92345098	9.67290319	1.92345098
Pd	-1.92345098	9.67290319	-1.92345098
Pd	-1.92345098	-9.67290319	1.92345098
Pd	1.92345098	-9.67290319	-1.92345098
Pd	1.92345098	1.92345098	9.67290319
Pd	1.92345098	-1.92345098	-9.67290319
Pd	-1.92345098	1.92345098	-9.67290319
Pd	-1.92345098	-1.92345098	9.67290319
Pd	9.67290319	-1.92345098	1.92345098
Pd	1.92345098	1.92345098	-9.67290319
Pd	-1.92345098	9.67290319	1.92345098
Pd	9.67290319	1.92345098	-1.92345098
Pd	-1.92345098	1.92345098	9.67290319
Pd	1.92345098	-9.67290319	1.92345098
Pd	1.92345098	9.67290319	-1.92345098
Pd	-1.92345098	-9.67290319	-1.92345098
Pd	1.92345098	-1.92345098	9.67290319
Pd	-9.67290319	1.92345098	1.92345098
Pd	-1.92345098	-1.92345098	-9.67290319

```
Pd   -9.67290319   -1.92345098   -1.92345098
Pd   9.627633697   3.851500975   0
Pd   9.627633697   -3.851500975   0
Pd   -9.627633697   3.851500975   0
Pd   -9.627633697   -3.851500975   0
Pd   0   9.627633697   3.851500975
Pd   0   9.627633697   -3.851500975
Pd   0   -9.627633697   3.851500975
Pd   0   -9.627633697   -3.851500975
Pd   3.851500975   0   9.627633697
Pd   3.851500975   0   -9.627633697
Pd   -3.851500975   0   -9.627633697
Pd   -3.851500975   0   9.627633697
Pd   9.627633697   0   3.851500975
Pd   0   3.851500975   -9.627633697
Pd   -3.851500975   9.627633697   0
Pd   9.627633697   0   -3.851500975
Pd   0   3.851500975   9.627633697
Pd   3.851500975   -9.627633697   0
Pd   3.851500975   9.627633697   0
Pd   -3.851500975   -9.627633697   0
Pd   0   -3.851500975   9.627633697
Pd   -9.627633697   0   3.851500975
Pd   0   -3.851500975   -9.627633697
Pd   -9.627633697   0   -3.851500975
```

NP Pd_{264} with 6Pd edge

```
-1244.654 eV
Pd   12.50000000000000   6.65888638839818   4.75266854847087
Pd   12.50000000000000   4.75266854847087   6.65888638839818
Pd   12.50000000000000   2.87631478606228   8.64849254083634
Pd   12.50000000000000   8.64849254083634   2.87631478606228
Pd   14.56105586291706   6.57599957128749   6.57599957128749
Pd   10.43894413708294   6.57599957128749   6.57599957128749
Pd   14.52792255395141   4.68503220831212   8.52954884353967
Pd   10.47207744604859   8.52954884353967   4.68503220831212
Pd   10.47207744604859   4.68503220831212   8.52954884353967
Pd   14.52792255395141   8.52954884353967   4.68503220831212
Pd   14.41452360438071   2.82501835324337   10.57710595543218
Pd   14.41452360438071   10.57710595543218   2.82501835324337
Pd   10.58547639561929   2.82501835324337   10.57710595543218
Pd   10.58547639561929   10.57710595543218   2.82501835324337
Pd   16.51302432884531   6.54208561624667   8.47825247830621
Pd   16.51302432884531   8.47825247830621   6.54208561624667
Pd   8.48697567115470   6.54208561624667   8.47825247830621
Pd   8.48697567115470   8.47825247830621   6.54208561624667
Pd   16.46362191405533   10.46056684860631   4.67727158906672
Pd   8.53637808594467   4.67727158906672   10.46056684860631
Pd   8.53637808594467   10.46056684860631   4.67727158906672
Pd   16.46362191405533   4.67727158906672   10.46056684860631
Pd   12.50000000000000   6.65470851688921   8.55439459643684
Pd   12.50000000000000   8.55439459643684   6.65470851688921
Pd   12.50000000000000   2.79256576313026   12.49556407147304
Pd   12.50000000000000   12.49556407147304   2.79256576313026
Pd   12.50000000000000   4.80147134956330   10.53633800596670
Pd   12.50000000000000   10.53633800596670   4.80147134956330
Pd   16.33805300044499   12.49589126737725   2.86332976708155
Pd   8.66194699955500   12.49589126737725   2.86332976708155
```

Pd	16.33805300044499	2.86332976708155	12.49589126737725
Pd	8.66194699955500	2.86332976708155	12.49589126737725
Pd	18.44698954553720	8.46518948188247	8.46518948188247
Pd	6.55301045446279	8.46518948188247	8.46518948188247
Pd	18.42087522666122	6.55171829579252	10.41771061528747
Pd	18.42087522666122	10.41771061528747	6.55171829579252
Pd	6.57912477333878	6.55171829579252	10.41771061528747
Pd	6.57912477333878	10.41771061528747	6.55171829579252
Pd	14.52005024943137	8.54876733691409	8.54876733691409
Pd	10.47994975056863	8.54876733691409	8.54876733691409
Pd	14.50335610475449	6.65281740196340	10.50231177140833
Pd	14.50335610475449	10.50231177140833	6.65281740196340
Pd	10.49664389524551	6.65281740196340	10.50231177140833
Pd	10.49664389524551	10.50231177140833	6.65281740196340
Pd	10.58106660452747	2.81122639422482	14.41547723464399
Pd	10.58106660452747	14.41547723464399	2.81122639422482
Pd	14.41893339547253	14.41547723464399	2.81122639422482
Pd	14.41893339547253	2.81122639422482	14.41547723464399
Pd	6.66924556055601	4.73296587827330	12.49343033418300
Pd	18.33075443944399	4.73296587827330	12.49343033418300
Pd	18.33075443944399	12.49343033418300	4.73296587827330
Pd	6.66924556055601	12.49343033418300	4.73296587827330
Pd	14.46518653738106	12.50301910077985	4.79633777061269
Pd	10.53481346261894	12.50301910077985	4.79633777061269
Pd	14.46518653738106	4.79633777061269	12.50301910077985
Pd	10.53481346261894	4.79633777061269	12.50301910077985
Pd	20.28850333785832	8.51096578238890	10.45259334950024
Pd	4.71149666214168	10.45259334950024	8.51096578238890
Pd	4.71149666214168	8.51096578238890	10.45259334950024
Pd	20.28850333785832	10.45259334950024	8.51096578238890
Pd	16.46569161397855	8.53990312551188	10.48666222769671
Pd	16.46569161397855	10.48666222769671	8.53990312551188
Pd	8.53430838602144	8.53990312551188	10.48666222769671
Pd	8.53430838602144	10.48666222769671	8.53990312551188
Pd	12.50000000000000	8.58133670351974	10.51597868894859
Pd	12.50000000000000	10.51597868894859	8.58133670351974
Pd	16.45392227778961	12.50244897288392	6.64022890369893
Pd	8.54607772221039	12.50244897288392	6.64022890369893
Pd	16.45392227778961	6.64022890369893	12.50244897288392
Pd	8.54607772221039	6.64022890369893	12.50244897288392
Pd	20.21856742669496	12.50017662135988	6.64045353483274
Pd	4.78143257330504	12.50017662135988	6.64045353483274
Pd	20.21856742669496	6.64045353483274	12.50017662135988
Pd	4.78143257330504	6.64045353483274	12.50017662135988
Pd	12.50000000000000	6.68377050715000	12.50482871187249
Pd	12.50000000000000	12.50482871187249	6.68377050715000
Pd	8.53379284597188	14.53129837570283	4.66756827498101
Pd	16.46620715402813	4.66756827498101	14.53129837570283
Pd	16.46620715402813	14.53129837570283	4.66756827498101
Pd	8.53379284597188	4.66756827498101	14.53129837570283
Pd	12.50000000000000	2.84847457682908	16.33633296058235
Pd	12.50000000000000	16.33633296058235	2.84847457682908
Pd	12.50000000000000	4.79114842341655	14.46613583877122
Pd	12.50000000000000	14.46613583877122	4.79114842341655
Pd	18.35279924108116	10.49856644510699	10.49856644510699
Pd	6.64720075891884	10.49856644510699	10.49856644510699
Pd	14.49387657404695	10.52117148862055	10.52117148862055
Pd	10.50612342595305	10.52117148862055	10.52117148862055
Pd	18.34298773170692	8.54459874829705	12.50496416070432
Pd	6.65701226829308	8.54459874829705	12.50496416070432

Pd	18.34298773170692	12.50496416070432	8.54459874829705
Pd	6.65701226829308	12.50496416070432	8.54459874829705
Pd	14.49864984166262	12.50661517504726	8.58044929597147
Pd	10.50135015833737	12.50661517504726	8.58044929597147
Pd	14.49864984166262	8.58044929597147	12.50661517504726
Pd	10.50135015833737	8.58044929597147	12.50661517504726
Pd	6.58070485307557	6.55821055599830	14.57280761719966
Pd	6.58070485307557	14.57280761719966	6.55821055599830
Pd	18.41929514692442	14.57280761719966	6.55821055599830
Pd	18.41929514692442	6.55821055599830	14.57280761719966
Pd	22.07597207723460	8.63119206584008	12.47260616504181
Pd	2.92402792276540	8.63119206584008	12.47260616504181
Pd	22.07597207723460	12.47260616504181	8.63119206584008
Pd	2.92402792276540	12.47260616504181	8.63119206584008
Pd	10.50067055262195	6.64906383596997	14.50745027412073
Pd	10.50067055262195	14.50745027412073	6.64906383596997
Pd	14.49932944737805	14.50745027412073	6.64906383596997
Pd	14.49932944737805	6.64906383596997	14.50745027412073
Pd	22.12672785942437	10.54551663309500	10.54551663309500
Pd	2.87327214057563	10.54551663309500	10.54551663309500
Pd	10.46542598399252	4.66407102335636	16.45877482899802
Pd	14.53457401600749	16.45877482899802	4.66407102335636
Pd	14.53457401600749	4.66407102335636	16.45877482899802
Pd	10.46542598399252	16.45877482899802	4.66407102335636
Pd	16.44951084162976	10.52454960316309	12.51037661390924
Pd	8.55048915837024	10.52454960316309	12.51037661390924
Pd	16.44951084162976	12.51037661390924	10.52454960316309
Pd	8.55048915837024	12.51037661390924	10.52454960316309
Pd	12.50000000000000	10.52652421562808	12.49885561215076
Pd	12.50000000000000	12.49885561215076	10.52652421562808
Pd	20.17412602220117	10.53588546623378	12.50265205047275
Pd	4.82587397779884	10.53588546623378	12.50265205047275
Pd	20.17412602220117	12.50265205047275	10.53588546623378
Pd	4.82587397779884	12.50265205047275	10.53588546623378
Pd	8.54754231826650	8.54931120623674	14.51464091458459
Pd	8.54754231826650	14.51464091458459	8.54931120623674
Pd	16.45245768173349	14.51464091458459	8.54931120623674
Pd	16.45245768173349	8.54931120623674	14.51464091458459
Pd	12.50000000000000	8.58166089864518	14.49965936019232
Pd	12.50000000000000	14.49965936019232	8.58166089864518
Pd	4.71391106098312	8.52423174650792	14.52759215394770
Pd	20.28608893901688	14.52759215394770	8.52423174650792
Pd	20.28608893901688	8.52423174650792	14.52759215394770
Pd	4.71391106098312	14.52759215394770	8.52423174650792
Pd	8.48274540954976	6.53376549324307	16.50619048203818
Pd	8.48274540954976	16.50619048203818	6.53376549324307
Pd	16.51725459045023	16.50619048203818	6.53376549324307
Pd	16.51725459045023	6.53376549324307	16.50619048203818
Pd	12.50000000000000	18.32996656874329	4.71620357431593
Pd	12.50000000000000	4.71620357431593	18.32996656874329
Pd	12.50000000000000	6.63003760070978	16.45592188303888
Pd	12.50000000000000	16.45592188303888	6.63003760070978
Pd	14.48094319429388	12.49939558347734	12.49939558347734
Pd	10.51905680570612	12.49939558347734	12.49939558347734
Pd	10.51268043857625	14.48622067515562	10.53567483896260
Pd	10.51268043857625	10.53567483896260	14.48622067515562
Pd	14.48731956142375	10.53567483896260	14.48622067515562
Pd	14.48731956142375	14.48622067515562	10.53567483896260
Pd	18.34374465653758	12.53294770548593	12.53294770548593
Pd	6.65625534346242	12.53294770548593	12.53294770548593

Pd	10.48548679875405	16.45309236470066	8.54939684788180
Pd	10.48548679875405	8.54939684788180	16.45309236470066
Pd	14.51451320124595	16.45309236470066	8.54939684788180
Pd	14.51451320124595	8.54939684788180	16.45309236470066
Pd	6.66018978197100	14.50480355996353	10.54836752176231
Pd	6.66018978197100	10.54836752176231	14.50480355996353
Pd	18.33981021802900	10.54836752176231	14.50480355996353
Pd	18.33981021802900	14.50480355996353	10.54836752176231
Pd	6.57472242264593	16.49219944452629	8.48880021662491
Pd	6.57472242264593	8.48880021662491	16.49219944452629
Pd	18.42527757735407	8.48880021662491	16.49219944452629
Pd	18.42527757735407	16.49219944452629	8.48880021662491
Pd	10.41606706481751	18.40603814794678	6.54968608146558
Pd	10.41606706481751	6.54968608146558	18.40603814794678
Pd	14.58393293518249	18.40603814794678	6.54968608146558
Pd	14.58393293518249	6.54968608146558	18.40603814794678
Pd	22.12555025815280	12.47298943336872	12.47298943336872
Pd	2.87444974184719	12.47298943336872	12.47298943336872
Pd	2.91020096086053	14.40666423142671	10.56809114424388
Pd	2.91020096086053	10.56809114424388	14.40666423142671
Pd	22.08979903913947	10.56809114424388	14.40666423142671
Pd	22.08979903913947	14.40666423142671	10.56809114424388
Pd	12.50000000000000	18.32500065254634	8.55227318629966
Pd	12.50000000000000	8.55227318629966	18.32500065254634
Pd	12.50000000000000	6.63113374829568	20.18797669103296
Pd	12.50000000000000	20.18797669103296	6.63113374829568
Pd	8.48449561696383	18.40359787236098	8.48614038241736
Pd	8.48449561696383	8.48614038241736	18.40359787236098
Pd	16.51550438303617	18.40359787236098	8.48614038241736
Pd	16.51550438303617	8.48614038241736	18.40359787236098
Pd	4.77014880849103	16.40411583288776	10.56324783217391
Pd	20.22985119150897	10.56324783217391	16.40411583288776
Pd	20.22985119150897	16.40411583288776	10.56324783217391
Pd	4.77014880849103	10.56324783217391	16.40411583288776
Pd	12.50000000000000	16.44806437719029	10.53609467866991
Pd	12.50000000000000	10.53609467866991	16.44806437719029
Pd	8.54852617456153	16.43301157809994	10.55129943537380
Pd	8.54852617456153	10.55129943537380	16.43301157809994
Pd	16.45147382543847	10.55129943537380	16.43301157809994
Pd	16.45147382543847	16.43301157809994	10.55129943537380
Pd	20.28755514810547	14.51883883241296	12.58567777042714
Pd	4.71244485189453	14.51883883241296	12.58567777042714
Pd	20.28755514810547	12.58567777042714	14.51883883241296
Pd	4.71244485189453	12.58567777042714	14.51883883241296
Pd	12.50000000000000	14.46977246790872	12.49966912932917
Pd	12.50000000000000	12.49966912932917	14.46977246790872
Pd	16.44192750528662	14.49458435628098	12.53977626063513
Pd	8.55807249471338	14.49458435628098	12.53977626063513
Pd	16.44192750528662	12.53977626063513	14.49458435628098
Pd	8.55807249471338	12.53977626063513	14.49458435628098
Pd	10.46135168711845	20.24468830298144	8.52277763578970
Pd	14.53864831288155	8.52277763578970	20.24468830298144
Pd	14.53864831288155	20.24468830298144	8.52277763578970
Pd	10.46135168711845	8.52277763578970	20.24468830298144
Pd	10.49098673051820	18.31384196397859	10.55225914799100
Pd	10.49098673051820	10.55225914799100	18.31384196397859
Pd	14.50901326948180	18.31384196397859	10.55225914799100
Pd	14.50901326948180	10.55225914799100	18.31384196397859
Pd	6.65269403678958	18.31649813659708	10.57020744682378
Pd	6.65269403678958	10.57020744682378	18.31649813659708

Pd	18.34730596321042	10.57020744682378	18.31649813659708
Pd	18.34730596321042	18.31649813659708	10.57020744682378
Pd	14.49732788111753	12.54106334968456	16.42308975617911
Pd	10.50267211888247	12.54106334968456	16.42308975617911
Pd	10.50267211888247	16.42308975617911	12.54106334968456
Pd	14.49732788111753	16.42308975617911	12.54106334968456
Pd	18.41557251863672	16.48133452439203	12.62943685008338
Pd	6.58442748136327	16.48133452439203	12.62943685008338
Pd	18.41557251863672	12.62943685008338	16.48133452439203
Pd	6.58442748136327	12.62943685008338	16.48133452439203
Pd	14.49110806380736	14.47290576852908	14.47290576852908
Pd	10.50889193619264	14.47290576852908	14.47290576852908
Pd	18.42189179852954	14.55648355344313	14.55648355344313
Pd	6.57810820147046	14.55648355344313	14.55648355344313
Pd	12.50000000000000	20.13229680568215	10.54696739138815
Pd	12.50000000000000	10.54696739138815	20.13229680568215
Pd	12.50000000000000	22.02037164422894	8.61897969532297
Pd	12.50000000000000	8.61897969532297	22.02037164422894
Pd	8.56735920675342	10.55980183623026	20.18547137300308
Pd	16.43264079324658	20.18547137300308	10.55980183623026
Pd	16.43264079324658	10.55980183623026	20.18547137300308
Pd	8.56735920675342	20.18547137300308	10.55980183623026
Pd	12.50000000000000	18.30502491657312	12.53810142079100
Pd	12.50000000000000	12.53810142079100	18.30502491657312
Pd	16.49869254132464	12.63117898909579	18.38325076301451
Pd	8.50130745867536	12.63117898909579	18.38325076301451
Pd	8.50130745867536	18.38325076301451	12.63117898909579
Pd	16.49869254132464	18.38325076301451	12.63117898909579
Pd	12.50000000000000	16.40204022526106	14.47823226781187
Pd	12.50000000000000	14.47823226781187	16.40204022526106
Pd	16.49240959919797	16.47664152230100	14.55712061634989
Pd	16.49240959919797	14.55712061634989	16.47664152230100
Pd	8.50759040080204	16.47664152230100	14.55712061634989
Pd	8.50759040080204	14.55712061634989	16.47664152230100
Pd	14.52422732389470	12.57260777543038	20.23452099244308
Pd	10.47577267610530	12.57260777543038	20.23452099244308
Pd	10.47577267610530	20.23452099244308	12.57260777543038
Pd	14.52422732389470	20.23452099244308	12.57260777543038
Pd	10.55737236887844	22.03183181686496	10.54500850421374
Pd	10.55737236887844	10.54500850421374	22.03183181686496
Pd	14.44262763112156	22.03183181686496	10.54500850421374
Pd	14.44262763112156	10.54500850421374	22.03183181686496
Pd	14.54600777115576	18.37488967354143	14.53902208821902
Pd	14.54600777115576	14.53902208821902	18.37488967354143
Pd	10.45399222884425	18.37488967354143	14.53902208821902
Pd	10.45399222884425	14.53902208821902	18.37488967354143
Pd	14.54008261039665	16.45022932181623	16.45022932181623
Pd	10.45991738960335	16.45022932181623	16.45022932181623
Pd	12.50000000000000	20.18367418461825	14.46704841287829
Pd	12.50000000000000	14.46704841287829	20.18367418461825
Pd	12.50000000000000	22.04047623229988	12.46319424959033
Pd	12.50000000000000	12.46319424959033	22.04047623229988
Pd	12.50000000000000	18.29837556010910	16.39399739885849
Pd	12.50000000000000	16.39399739885849	18.29837556010910

NP Pd$_{293}$ with 7Pd edge

-1383.932 eV

Pd	12.50000000000000	2.83890408365260	6.70739394896844
Pd	12.50000000000000	6.70739394896844	2.83890408365260

Pd	12.50000000000000	4.72334928697508	4.72334928697508
Pd	14.52210510380999	4.66287893319635	6.59791914064646
Pd	10.47789489619001	6.59791914064646	4.66287893319635
Pd	10.47789489619001	4.66287893319635	6.59791914064646
Pd	14.52210510380999	6.59791914064646	4.66287893319635
Pd	14.42948939747383	2.77451054015171	8.62557517558472
Pd	10.57051060252617	8.62557517558472	2.77451054015171
Pd	10.57051060252617	2.77451054015171	8.62557517558472
Pd	14.42948939747383	8.62557517558472	2.77451054015171
Pd	12.50000000000000	6.63720733247141	6.63720733247141
Pd	16.49530284912282	6.54057684036444	6.54057684036445
Pd	8.50469715087719	6.54057684036444	6.54057684036444
Pd	16.34827594435721	10.54146589989172	2.76842158340566
Pd	8.65172405564278	2.76842158340566	10.54146589989172
Pd	8.65172405564278	10.54146589989172	2.76842158340566
Pd	16.34827594435721	2.76842158340566	10.54146589989172
Pd	12.50000000000000	4.75692998033621	8.60751888715409
Pd	12.50000000000000	8.60751888715409	4.75692998033621
Pd	12.50000000000000	2.73707360941691	10.55453027147991
Pd	12.50000000000000	10.55453027147991	2.73707360941691
Pd	16.45708411943827	4.64421469950412	8.51576389729850
Pd	16.45708411943827	8.51576389729850	4.64421469950412
Pd	8.54291588056173	4.64421469950412	8.51576389729850
Pd	8.54291588056173	8.51576389729850	4.64421469950412
Pd	14.49905128707638	8.56238457133667	6.63647442300656
Pd	10.50094871292363	6.63647442300656	8.56238457133667
Pd	10.50094871292363	8.56238457133667	6.63647442300656
Pd	14.49905128707638	6.63647442300656	8.56238457133667
Pd	14.45421956909770	4.73457814324574	10.55127774458946
Pd	14.45421956909770	10.55127774458946	4.73457814324574
Pd	10.54578043090230	4.73457814324574	10.55127774458946
Pd	10.54578043090230	10.55127774458946	4.73457814324574
Pd	18.43248903798381	8.47433357357796	6.52902858441298
Pd	18.43248903798381	6.52902858441298	8.47433357357796
Pd	6.56751096201619	8.47433357357796	6.52902858441298
Pd	6.56751096201619	6.52902858441298	8.47433357357796
Pd	18.36668369867696	10.45903827760761	4.64157119626795
Pd	6.63331630132304	4.64157119626795	10.45903827760761
Pd	6.63331630132304	10.45903827760761	4.64157119626795
Pd	18.36668369867696	4.64157119626795	10.45903827760761
Pd	18.25813203817270	12.48551567129316	2.80546833957209
Pd	6.74186796182730	12.48551567129316	2.80546833957209
Pd	18.25813203817270	2.80546833957209	12.48551567129316
Pd	6.74186796182730	2.80546833957209	12.48551567129316
Pd	14.43395469551186	12.49331318357964	2.72600539339179
Pd	10.56604530448814	12.49331318357964	2.72600539339179
Pd	14.43395469551186	2.72600539339179	12.49331318357964
Pd	10.56604530448814	2.72600539339179	12.49331318357964
Pd	12.50000000000000	6.67779442827615	10.54998518889940
Pd	12.50000000000000	10.54998518889940	6.67779442827615
Pd	16.44675140216343	6.63179669319991	10.51249062639935
Pd	8.55324859783657	10.51249062639935	6.63179669319991
Pd	8.55324859783657	6.63179669319991	10.51249062639935
Pd	16.44675140216343	10.51249062639935	6.63179669319991
Pd	20.28486882768745	6.57580615005155	10.45919015413487
Pd	4.71513117231255	10.45919015413487	6.57580615005155
Pd	4.71513117231255	6.57580615005155	10.45919015413487
Pd	20.28486882768745	10.45919015413487	6.57580615005155
Pd	8.64402462998066	14.42338101706766	2.73346059556809
Pd	16.35597537001934	2.73346059556809	14.42338101706766

Pd	16.35597537001934	14.42338101706766	2.73346059556809
Pd	8.64402462998066	2.73346059556809	14.42338101706766
Pd	12.50000000000000	8.58419161330162	8.58419161330162
Pd	16.47089448219871	8.54218873301510	8.54218873301510
Pd	8.52910551780131	8.54218873301510	8.54218873301510
Pd	12.50000000000000	4.71833789101143	12.51093626692542
Pd	12.50000000000000	12.51093626692542	4.71833789101143
Pd	16.38889987261860	12.50697913088905	4.73573831276295
Pd	8.61110012738139	12.50697913088905	4.73573831276295
Pd	16.38889987261860	4.73573831276295	12.50697913088905
Pd	8.61110012738139	4.73573831276295	12.50697913088905
Pd	20.30663466794282	8.49890581744510	8.49890581744510
Pd	4.69336533205718	8.49890581744510	8.49890581744510
Pd	20.22729997831307	4.70188908930764	12.49394358434504
Pd	4.77270002168693	4.70188908930764	12.49394358434504
Pd	20.22729997831307	12.49394358434504	4.70188908930764
Pd	4.77270002168693	12.49394358434504	4.70188908930764
Pd	12.50000000000000	2.70711325176187	14.42679064491596
Pd	12.50000000000000	14.42679064491596	2.70711325176187
Pd	10.55068193839137	2.72097190139978	16.33371586757102
Pd	14.44931806160863	16.33371586757102	2.72097190139978
Pd	14.44931806160863	2.72097190139978	16.33371586757102
Pd	10.55068193839137	16.33371586757102	2.72097190139978
Pd	14.48950434159233	8.59822456117825	10.54183512441839
Pd	14.48950434159233	10.54183512441839	8.59822456117825
Pd	10.51049565840767	8.59822456117825	10.54183512441839
Pd	10.51049565840767	10.54183512441839	8.59822456117825
Pd	14.47305492139405	12.52673964575540	6.68242470038053
Pd	10.52694507860595	12.52673964575540	6.68242470038053
Pd	14.47305492139405	6.68242470038053	12.52673964575540
Pd	10.52694507860595	6.68242470038053	12.52673964575540
Pd	18.36478563628300	10.51312232410102	8.55702410584403
Pd	6.63521436371700	8.55702410584403	10.51312232410102
Pd	6.63521436371700	10.51312232410102	8.55702410584403
Pd	18.36478563628300	8.55702410584403	10.51312232410102
Pd	18.34305299003045	6.62836857588097	12.51778333312500
Pd	6.65694700996955	6.62836857588097	12.51778333312500
Pd	18.34305299003045	12.51778333312500	6.62836857588097
Pd	6.65694700996955	12.51778333312500	6.62836857588097
Pd	22.13258952992662	8.61362304695649	10.53373703137827
Pd	2.86741047007338	10.53373703137827	8.61362304695649
Pd	2.86741047007338	8.61362304695649	10.53373703137827
Pd	22.13258952992662	10.53373703137827	8.61362304695649
Pd	22.08861687069510	6.69609465501425	12.47304397550635
Pd	2.91138312930491	6.69609465501425	12.47304397550635
Pd	22.08861687069510	12.47304397550635	6.69609465501425
Pd	2.91138312930491	12.47304397550635	6.69609465501425
Pd	10.54718360532357	4.71715337010469	14.45890208339727
Pd	10.54718360532357	14.45890208339727	4.71715337010469
Pd	14.45281639467643	14.45890208339727	4.71715337010469
Pd	14.45281639467643	4.71715337010469	14.45890208339727
Pd	6.61916927725552	14.52323219227005	4.62836297310301
Pd	18.38083072274448	4.62836297310301	14.52323219227005
Pd	18.38083072274448	14.52323219227005	4.62836297310301
Pd	6.61916927725552	4.62836297310301	14.52323219227005
Pd	12.50000000000000	10.54718660080582	10.54718660080582
Pd	16.44522251975592	10.55071101632832	10.55071101632832
Pd	8.55477748024408	10.55071101632832	10.55071101632832
Pd	20.20882502749703	10.54843535376156	10.54843535376156
Pd	4.79117497250296	10.54843535376156	10.54843535376156

Pd	12.50000000000000	2.77369410624348	18.23464810150190
Pd	12.50000000000000	18.23464810150190	2.77369410624348
Pd	12.50000000000000	8.62765995811627	12.53350715890390
Pd	12.50000000000000	12.53350715890390	8.62765995811627
Pd	16.45069820152309	8.60301812776471	12.53378688688732
Pd	8.54930179847691	8.60301812776471	12.53378688688732
Pd	16.45069820152309	12.53378688688732	8.60301812776471
Pd	8.54930179847691	12.53378688688732	8.60301812776471
Pd	12.50000000000000	6.68276623513917	14.50227143585548
Pd	12.50000000000000	14.50227143585548	6.68276623513917
Pd	8.56240686397603	6.63373239358358	14.51258512327222
Pd	16.43759313602397	14.51258512327222	6.63373239358358
Pd	16.43759313602397	6.63373239358358	14.51258512327222
Pd	8.56240686397603	14.51258512327222	6.63373239358358
Pd	20.19464523602955	8.60378560474697	12.52061127090692
Pd	4.80535476397045	8.60378560474697	12.52061127090692
Pd	12.50000000000000	4.70549793747114	16.38958732763741
Pd	12.50000000000000	16.38958732763741	4.70549793747114
Pd	20.19464523602955	12.52061127090692	8.60378560474697
Pd	4.80535476397045	12.52061127090692	8.60378560474697
Pd	8.52548672487331	4.59771020616672	16.44595805863236
Pd	8.52548672487331	16.44595805863236	4.59771020616672
Pd	16.47451327512670	16.44595805863236	4.59771020616672
Pd	16.47451327512670	4.59771020616672	16.44595805863236
Pd	4.71271650798970	6.58311069098517	14.52571503222097
Pd	20.28728349201030	14.52571503222097	6.58311069098517
Pd	20.28728349201030	6.58311069098517	14.52571503222097
Pd	4.71271650798970	14.52571503222097	6.58311069098517
Pd	14.48527934946675	10.56069450747651	12.52512656821775
Pd	10.51472065053325	10.56069450747651	12.52512656821775
Pd	14.48527934946675	12.52512656821775	10.56069450747651
Pd	10.51472065053325	12.52512656821775	10.56069450747651
Pd	18.36196893027786	10.59411999301451	12.55092462824107
Pd	6.63803106972213	10.59411999301451	12.55092462824107
Pd	18.36196893027786	12.55092462824107	10.59411999301451
Pd	6.63803106972213	12.55092462824107	10.59411999301451
Pd	10.49931446404418	16.44519354436044	6.62989095340349
Pd	14.50068553595583	6.62989095340349	16.44519354436044
Pd	14.50068553595583	16.44519354436044	6.62989095340349
Pd	10.49931446404418	6.62989095340349	16.44519354436044
Pd	22.15033510721248	10.54488320663052	12.47254739747526
Pd	2.84966489278752	10.54488320663052	12.47254739747526
Pd	22.15033510721248	12.47254739747526	10.54488320663052
Pd	2.84966489278752	12.47254739747526	10.54488320663052
Pd	6.58568690187306	16.47218873533722	6.53469594933962
Pd	6.58568690187306	6.53469594933962	16.47218873533722
Pd	18.41431309812694	16.47218873533722	6.53469594933962
Pd	18.41431309812694	6.53469594933962	16.47218873533722
Pd	10.50778307919621	8.63160995454610	14.51587247427438
Pd	10.50778307919621	14.51587247427438	8.63160995454610
Pd	14.49221692080379	14.51587247427438	8.63160995454610
Pd	14.49221692080379	8.63160995454610	14.51587247427438
Pd	6.65756778290377	14.51736826774472	8.61920171978607
Pd	18.34243221709623	8.61920171978607	14.51736826774472
Pd	18.34243221709623	14.51736826774472	8.61920171978607
Pd	6.65756778290377	8.61920171978607	14.51736826774472
Pd	10.44601806608142	4.60112997748791	18.34851752041173
Pd	14.55398193391858	18.34851752041173	4.60112997748791
Pd	14.55398193391858	4.60112997748791	18.34851752041173
Pd	10.44601806608142	18.34851752041173	4.60112997748791

Pd	2.88984926160560	8.62649360024630	14.42293975431569
Pd	22.11015073839440	14.42293975431569	8.62649360024630
Pd	22.11015073839440	8.62649360024630	14.42293975431569
Pd	2.88984926160560	14.42293975431569	8.62649360024630
Pd	12.50000000000000	12.50000000000000	12.50000000000000
Pd	12.50000000000000	14.50252930322072	10.58698299640070
Pd	12.50000000000000	10.58698299640070	14.50252930322072
Pd	16.45628127880016	12.55153334600278	12.55153334600278
Pd	8.54371872119984	12.55153334600278	12.55153334600278
Pd	8.54605717148437	14.51145762158526	10.62201851669943
Pd	8.54605717148437	10.62201851669943	14.51145762158526
Pd	16.45394282851563	10.62201851669943	14.51145762158526
Pd	16.45394282851563	14.51145762158526	10.62201851669943
Pd	12.50000000000000	16.46416399308976	8.63753225594503
Pd	12.50000000000000	8.63753225594503	16.46416399308976
Pd	8.54263954343490	16.44238917165680	8.62850020367402
Pd	8.54263954343490	8.62850020367402	16.44238917165680
Pd	16.45736045656510	8.62850020367402	16.44238917165680
Pd	16.45736045656510	16.44238917165680	8.62850020367402
Pd	12.50000000000000	18.30752358886850	6.62761182319365
Pd	12.50000000000000	6.62761182319365	18.30752358886850
Pd	20.32334578190800	12.58503114255238	12.58503114255238
Pd	4.67665421809199	12.58503114255238	12.58503114255238
Pd	4.68765497203949	14.52629849111286	10.65243492039796
Pd	4.68765497203949	10.65243492039796	14.52629849111286
Pd	20.31234502796052	10.65243492039796	14.52629849111286
Pd	20.31234502796052	14.52629849111286	10.65243492039796
Pd	8.49678012795350	18.37685367322839	6.52298508723383
Pd	8.49678012795350	6.52298508723383	18.37685367322839
Pd	16.50321987204650	6.52298508723383	18.37685367322839
Pd	16.50321987204650	18.37685367322839	6.52298508723383
Pd	4.76965110221294	16.40840980552603	8.61554818285786
Pd	4.76965110221294	8.61554818285786	16.40840980552603
Pd	20.23034889778706	8.61554818285786	16.40840980552603
Pd	20.23034889778706	16.40840980552603	8.61554818285786
Pd	12.50000000000000	20.16190261043083	4.68785407257003
Pd	12.50000000000000	4.68785407257003	20.16190261043083
Pd	10.49104913778885	16.44998356435021	10.63605802527747
Pd	10.49104913778885	10.63605802527747	16.44998356435021
Pd	14.50895086221115	16.44998356435021	10.63605802527747
Pd	14.50895086221115	10.63605802527747	16.44998356435021
Pd	6.56306163887946	10.70360202893730	16.48547307814924
Pd	18.43693836112054	16.48547307814924	10.70360202893730
Pd	18.43693836112054	10.70360202893730	16.48547307814924
Pd	6.56306163887946	16.48547307814924	10.70360202893730
Pd	10.46426759812056	20.21213153677897	6.57776759722325
Pd	14.53573240187944	6.57776759722325	20.21213153677897
Pd	14.53573240187944	20.21213153677897	6.57776759722325
Pd	10.46426759812056	6.57776759722325	20.21213153677897
Pd	14.49500864829736	14.48667272410528	12.54623863886153
Pd	10.50499135170264	14.48667272410528	12.54623863886153
Pd	14.49500864829736	12.54623863886153	14.48667272410528
Pd	10.50499135170264	12.54623863886153	14.48667272410528
Pd	18.44117073572249	14.55723349803822	12.62875732125147
Pd	6.55882926427751	14.55723349803822	12.62875732125147
Pd	18.44117073572249	12.62875732125147	14.55723349803822
Pd	6.55882926427751	12.62875732125147	14.55723349803822
Pd	10.48476390231988	8.62951181528183	18.31108869730830
Pd	14.51523609768012	18.31108869730830	8.62951181528183
Pd	14.51523609768012	8.62951181528183	18.31108869730830

```
Pd   10.48476390231988   18.31108869730830   8.62951181528183
Pd   6.65472042390217   8.61850077830324   18.30944504364661
Pd   6.65472042390217   18.30944504364661   8.61850077830324
Pd   18.34527957609783   18.30944504364661   8.61850077830324
Pd   18.34527957609783   8.61850077830324   18.30944504364661
Pd   12.50000000000000   16.41780040787036   12.56090913987504
Pd   12.50000000000000   12.56090913987504   16.41780040787036
Pd   16.50967074542967   16.48187602507538   12.63928654735758
Pd   8.49032925457033   16.48187602507538   12.63928654735758
Pd   16.50967074542967   12.63928654735758   16.48187602507538
Pd   8.49032925457033   12.63928654735758   16.48187602507538
Pd   12.50000000000000   18.32354498845393   10.63174368560415
Pd   12.50000000000000   10.63174368560415   18.32354498845393
Pd   8.48417833553382   18.39286914631994   10.71101562362487
Pd   16.51582166446618   10.71101562362487   18.39286914631994
Pd   16.51582166446618   18.39286914631994   10.71101562362487
Pd   8.48417833553382   10.71101562362487   18.39286914631994
Pd   12.50000000000000   20.11936770270231   8.61944779318013
Pd   12.50000000000000   8.61944779318013   20.11936770270231
Pd   8.56644721895069   20.16326788290159   8.61497632682788
Pd   8.56644721895069   8.61497632682788   20.16326788290159
Pd   16.43355278104931   20.16326788290159   8.61497632682788
Pd   16.43355278104931   8.61497632682788   20.16326788290159
Pd   12.50000000000000   21.99812309023989   6.67786529122850
Pd   12.50000000000000   6.67786529122850   21.99812309023989
Pd   12.50000000000000   14.46620102840074   14.46620102840074
Pd   16.49178348378250   14.54954751894974   14.54954751894974
Pd   8.50821651621750   14.54954751894974   14.54954751894974
Pd   10.46908110110949   20.23611170422661   10.65182326419040
Pd   10.46908110110949   10.65182326419040   20.23611170422661
Pd   14.53091889889052   20.23611170422661   10.65182326419040
Pd   14.53091889889052   10.65182326419040   20.23611170422661
Pd   14.54059659882075   16.45095731277026   14.53308932911233
Pd   14.54059659882075   14.53308932911233   16.45095731277026
Pd   10.45940340117924   16.45095731277026   14.53308932911233
Pd   10.45940340117924   14.53308932911233   16.45095731277026
Pd   14.57038378190326   12.63131100969671   18.39386523179131
Pd   10.42961621809674   12.63131100969671   18.39386523179131
Pd   10.42961621809674   18.39386523179131   12.63131100969671
Pd   14.57038378190326   18.39386523179131   12.63131100969671
Pd   10.55688939223931   22.00715499654767   8.60729845862277
Pd   14.44311060776070   8.60729845862277   22.00715499654767
Pd   14.44311060776070   22.00715499654767   8.60729845862277
Pd   10.55688939223931   8.60729845862277   22.00715499654767
Pd   12.50000000000000   22.02808433460336   10.54083961444867
Pd   12.50000000000000   20.19457602300773   12.55104336439696
Pd   12.50000000000000   18.30402573771150   14.46607187499671
Pd   12.50000000000000   16.38183452047801   16.38183452047801
Pd   12.50000000000000   14.46607187499671   18.30402573771150
Pd   12.50000000000000   12.55104336439696   20.19457602300773
Pd   12.50000000000000   10.54083961444867   22.02808433460336
```

2CO molecule on 3Pd edge of Pd_{201} NP

```
-971.829 eV
Pd   12.5   12.4776   12.4776
Pd   12.5   10.5124   10.5124
Pd   10.52   12.4904   10.514
Pd   10.52   10.514   12.4904
Pd   12.5   10.51   14.459
```

Pd	12.5	14.459	10.51
Pd	14.48	12.4904	10.514
Pd	10.534	12.4946	14.4713
Pd	10.534	14.4713	12.4946
Pd	14.48	10.514	12.4904
Pd	12.5	14.4595	14.4595
Pd	14.466	12.4946	14.4713
Pd	14.466	14.4713	12.4946
Pd	16.3883	12.5038	12.5038
Pd	12.5	16.3846	12.4765
Pd	12.5	12.4765	16.3846
Pd	8.6117	12.5038	12.5038
Pd	12.5	8.5985	12.4906
Pd	12.5	12.4906	8.5985
Pd	14.4952	16.4225	10.4943
Pd	10.5429	16.4325	14.5067
Pd	16.42	10.5014	14.5006
Pd	16.42	14.5006	10.5014
Pd	10.5429	14.5067	16.4325
Pd	14.4952	10.4943	16.4225
Pd	16.4013	14.5038	14.5038
Pd	16.4283	10.4991	10.4991
Pd	10.5048	16.4225	10.4943
Pd	14.4571	16.4325	14.5067
Pd	10.5048	10.4943	16.4225
Pd	14.4571	14.5067	16.4325
Pd	10.4889	8.5676	10.4921
Pd	14.5018	8.568	14.4966
Pd	8.5717	10.4991	10.4991
Pd	8.5987	14.5038	14.5038
Pd	10.4889	10.4921	8.5676
Pd	14.5018	14.4966	8.568
Pd	8.58	10.5014	14.5006
Pd	8.58	14.5006	10.5014
Pd	10.4982	8.568	14.4966
Pd	14.5111	8.5676	10.4921
Pd	14.5111	10.4921	8.5676
Pd	10.4982	14.4966	8.568
Pd	8.567	8.5673	12.5049
Pd	16.4142	16.4188	12.5052
Pd	8.5858	16.4188	12.5052
Pd	16.433	8.5673	12.5049
Pd	8.567	12.5049	8.5673
Pd	16.4142	12.5052	16.4188
Pd	16.433	12.5049	8.5673
Pd	8.5858	12.5052	16.4188
Pd	12.5	8.5701	8.5701
Pd	12.5	16.4956	16.4956
Pd	12.5	8.5676	16.4257
Pd	12.5	16.4257	8.5676
Pd	12.5	14.4625	6.7193
Pd	12.5	14.4621	18.3119
Pd	12.5	6.7193	14.4625
Pd	12.5	18.3119	14.4621
Pd	6.724	12.5037	14.4664
Pd	18.276	12.5037	14.4664
Pd	14.4661	6.7204	12.4972
Pd	14.4569	18.282	12.4916
Pd	14.4569	12.4916	18.282
Pd	14.4661	12.4972	6.7204

Pd	6.724	14.4664	12.5037
Pd	18.276	14.4664	12.5037
Pd	12.5	10.5301	6.7207
Pd	12.5	10.5274	18.281
Pd	12.5	6.7207	10.5301
Pd	12.5	18.281	10.5274
Pd	6.7234	12.5044	10.5399
Pd	18.2766	12.5044	10.5399
Pd	10.5339	6.7204	12.4972
Pd	10.5431	18.282	12.4916
Pd	10.5431	12.4916	18.282
Pd	10.5339	12.4972	6.7204
Pd	6.7234	10.5399	12.5044
Pd	18.2766	10.5399	12.5044
Pd	16.4803	16.4882	16.4882
Pd	16.4997	16.4964	8.5002
Pd	8.5197	16.4882	16.4882
Pd	16.4997	8.5002	16.4964
Pd	8.5003	8.5002	16.4964
Pd	16.5161	8.492	8.492
Pd	8.5003	16.4964	8.5002
Pd	8.4839	8.492	8.492
Pd	16.4589	18.3808	14.5135
Pd	14.5344	16.481	18.3974
Pd	18.3929	14.539	16.4668
Pd	18.3929	16.4668	14.539
Pd	16.4589	14.5135	18.3808
Pd	14.5344	18.3974	16.481
Pd	6.6071	16.4668	14.539
Pd	14.5384	6.6018	16.4697
Pd	16.4761	6.5986	14.5414
Pd	6.6071	14.539	16.4668
Pd	16.4761	14.5414	6.5986
Pd	14.5384	16.4697	6.6018
Pd	18.3965	16.4635	10.463
Pd	10.4656	18.3974	16.481
Pd	16.4713	18.3861	10.4629
Pd	18.3965	10.463	16.4635
Pd	16.4713	10.4629	18.3861
Pd	10.4656	16.481	18.3974
Pd	16.4811	6.6024	10.4613
Pd	10.4616	16.4697	6.6018
Pd	6.6035	10.463	16.4635
Pd	6.6035	16.4635	10.463
Pd	16.4811	10.4613	6.6024
Pd	10.4616	6.6018	16.4697
Pd	18.3981	8.5273	14.5376
Pd	8.5411	14.5135	18.3808
Pd	14.5407	18.3951	8.5233
Pd	8.5411	18.3808	14.5135
Pd	14.5407	8.5233	18.3951
Pd	18.3981	14.5376	8.5273
Pd	8.5239	6.5986	14.5414
Pd	14.5517	8.5266	6.6016
Pd	6.6019	14.5376	8.5273
Pd	6.6019	8.5273	14.5376
Pd	8.5239	14.5414	6.5986
Pd	14.5517	6.6016	8.5266
Pd	8.5287	18.3861	10.4629
Pd	10.4593	8.5233	18.3951

Pd	18.396	10.4676	8.5325
Pd	18.396	8.5325	10.4676
Pd	8.5287	10.4629	18.3861
Pd	10.4593	18.3951	8.5233
Pd	6.604	8.5325	10.4676
Pd	8.5189	10.4613	6.6024
Pd	10.4483	6.6016	8.5266
Pd	8.5189	6.6024	10.4613
Pd	10.4483	8.5266	6.6016
Pd	6.604	10.4676	8.5325
Pd	12.5	4.7215	12.5005
Pd	4.7246	12.5021	12.5021
Pd	12.5	12.5005	4.7215
Pd	12.5	20.2919	12.4787
Pd	20.2754	12.5021	12.5021
Pd	12.5	12.4787	20.2919
Pd	14.434	20.2418	10.561
Pd	10.5811	20.2335	14.4191
Pd	20.2486	10.5726	14.4244
Pd	20.2486	14.4244	10.5726
Pd	10.5811	14.4191	20.2335
Pd	14.434	10.561	20.2418
Pd	20.2494	14.4266	14.4266
Pd	20.2438	10.5771	10.5771
Pd	10.566	20.2418	10.561
Pd	14.4189	20.2335	14.4191
Pd	10.566	10.561	20.2418
Pd	14.4189	14.4191	20.2335
Pd	10.5688	4.7569	10.5756
Pd	14.4279	4.7546	14.426
Pd	4.7562	10.5771	10.5771
Pd	4.7506	14.4266	14.4266
Pd	10.5688	10.5756	4.7569
Pd	14.4279	14.426	4.7546
Pd	4.7514	10.5726	14.4244
Pd	4.7514	14.4244	10.5726
Pd	10.5721	4.7546	14.426
Pd	14.4312	4.7569	10.5756
Pd	14.4312	10.5756	4.7569
Pd	10.5721	14.426	4.7546
Pd	6.6673	12.5023	6.6692
Pd	12.5	6.68	6.68
Pd	6.6673	6.6692	12.5023
Pd	18.3327	12.5023	6.6692
Pd	6.664	12.4934	18.3085
Pd	18.336	12.4934	18.3085
Pd	12.5	6.6692	18.3234
Pd	12.5	18.3234	6.6692
Pd	12.5	18.5352	18.5352
Pd	6.664	18.3085	12.4934
Pd	18.3327	6.6692	12.5023
Pd	18.336	18.3085	12.4934
Pd	8.6654	4.7972	12.5018
Pd	4.7963	12.5018	8.6694
Pd	12.5	8.6655	4.8068
Pd	4.7963	8.6694	12.5018
Pd	12.5	4.8068	8.6655
Pd	8.6654	12.5018	4.7972
Pd	16.3346	4.7972	12.5018
Pd	4.7895	12.4982	16.3215

```
Pd   12.5   16.3303   4.7921
Pd   4.7895   16.3215   12.4982
Pd   12.5   4.7921   16.3303
Pd   16.3346   12.5018   4.7972
Pd   8.6514   20.1943   12.4861
Pd   20.2037   12.5018   8.6694
Pd   12.5   8.6577   20.2002
Pd   20.2037   8.6694   12.5018
Pd   12.5   20.2002   8.6577
Pd   8.6514   12.4861   20.1943
Pd   16.3486   20.1943   12.4861
Pd   20.2105   12.4982   16.3215
Pd   12.5   16.4119   20.2391
Pd   20.2105   16.3215   12.4982
Pd   12.5   20.2391   16.4119
Pd   16.3486   12.4861   20.1943
C   12.5   18.3642   20.5723
C   12.5   20.5723   18.3642
O   12.5   19.063   21.519
O   12.5   21.519   19.063
```

3CO molecule on 4Pd edge of Pd_{314} NP

```
-1539.927 eV
Pd   14.47232912499992   12.50000000000000   12.50000000000000
Pd   10.52767087500008   12.50000000000000   12.50000000000000
Pd   12.50000000000000   14.47232912499992   12.50000000000000
Pd   12.50000000000000   10.52767087500008   12.50000000000000
Pd   12.50000000000000   12.50000000000000   14.47232912499992
Pd   12.50000000000000   12.50000000000000   10.52767087500008
Pd   14.47842099999992   14.47842099999992   14.47842099999992
Pd   14.47842099999992   10.52157900000008   10.52157900000008
Pd   10.52157900000008   14.47842099999992   10.52157900000008
Pd   10.52157900000008   10.52157900000008   14.47842099999992
Pd   14.47842099999992   10.52157900000008   14.47842099999992
Pd   14.47842099999992   14.47842099999992   10.52157900000008
Pd   10.52157900000008   14.47842099999992   14.47842099999992
Pd   10.52157900000008   10.52157900000008   10.52157900000008
Pd   16.43032219999991   14.49096055000005   12.50000000000000
Pd   16.43032219999991   10.50903944999994   12.50000000000000
Pd   8.56967780000008   14.49096055000005   12.50000000000000
Pd   8.56967780000008   10.50903944999994   12.50000000000000
Pd   12.50000000000000   16.43032219999991   14.49096055000005
Pd   12.50000000000000   16.43032219999991   10.50903944999994
Pd   12.50000000000000   8.56967780000008   14.49096055000005
Pd   12.50000000000000   8.56967780000008   10.50903944999994
Pd   14.49096055000005   12.50000000000000   16.43032219999991
Pd   14.49096055000005   12.50000000000000   8.56967780000008
Pd   10.50903944999994   12.50000000000000   8.56967780000008
Pd   10.50903944999994   12.50000000000000   16.43032219999991
Pd   16.43032219999991   12.50000000000000   14.49096055000005
Pd   12.50000000000000   14.49096055000005   8.56967780000008
Pd   10.50903944999994   16.43032219999991   12.50000000000000
Pd   16.43032219999991   12.50000000000000   10.50903944999994
Pd   12.50000000000000   14.49096055000005   16.43032219999991
Pd   14.49096055000005   8.56967780000008   12.50000000000000
Pd   14.49096055000005   16.43032219999991   12.50000000000000
Pd   10.50903944999994   8.56967780000008   12.50000000000000
Pd   12.50000000000000   10.50903944999994   16.43032219999991
```

Pd	8.56967780000008	12.50000000000000	14.49096055000005
Pd	12.50000000000000	10.50903944999994	8.56967780000008
Pd	8.56967780000008	12.50000000000000	10.50903944999994
Pd	18.33054834999999	12.50000000000000	12.50000000000000
Pd	6.66945165000001	12.50000000000000	12.50000000000000
Pd	12.50000000000000	18.33054834999999	12.50000000000000
Pd	12.50000000000000	6.66945165000001	12.50000000000000
Pd	12.50000000000000	12.50000000000000	18.33054834999999
Pd	12.50000000000000	12.50000000000000	6.66945165000001
Pd	16.45696470000004	16.45696470000004	14.51544027500002
Pd	16.45696470000004	8.54303529999996	10.48455972499998
Pd	8.54303529999996	16.45696470000004	10.48455972499998
Pd	8.54303529999996	8.54303529999996	14.51544027500002
Pd	14.51544027500002	16.45696470000004	16.45696470000004
Pd	10.48455972499998	16.45696470000004	8.54303529999996
Pd	10.48455972499998	8.54303529999996	16.45696470000004
Pd	14.51544027500002	8.54303529999996	8.54303529999996
Pd	16.45696470000004	14.51544027500002	16.45696470000004
Pd	16.45696470000004	10.48455972499998	8.54303529999996
Pd	8.54303529999996	14.51544027500002	8.54303529999996
Pd	8.54303529999996	10.48455972499998	16.45696470000004
Pd	16.45696470000004	10.48455972499998	16.45696470000004
Pd	14.51544027500002	16.45696470000004	8.54303529999996
Pd	8.54303529999996	16.45696470000004	14.51544027500002
Pd	16.45696470000004	14.51544027500002	8.54303529999996
Pd	10.48455972499998	16.45696470000004	16.45696470000004
Pd	16.45696470000004	8.54303529999996	14.51544027500002
Pd	16.45696470000004	16.45696470000004	10.48455972499998
Pd	8.54303529999996	8.54303529999996	10.48455972499998
Pd	14.51544027500002	8.54303529999996	16.45696470000004
Pd	8.54303529999996	14.51544027500002	16.45696470000004
Pd	10.48455972499998	8.54303529999996	8.54303529999996
Pd	8.54303529999996	10.48455972499998	8.54303529999996
Pd	18.35250092499994	14.50416267500003	14.50416267500003
Pd	18.35250092499994	10.49583732499997	10.49583732499997
Pd	6.64749907500006	14.50416267500003	10.49583732499997
Pd	6.64749907500006	10.49583732499997	14.50416267500003
Pd	14.50416267500003	18.35250092499994	14.50416267500003
Pd	10.49583732499997	18.35250092499994	10.49583732499997
Pd	10.49583732499997	6.64749907500006	14.50416267500003
Pd	14.50416267500003	6.64749907500006	10.49583732499997
Pd	14.50416267500003	14.50416267500003	18.35250092499994
Pd	14.50416267500003	10.49583732499997	6.64749907500006
Pd	10.49583732499997	14.50416267500003	6.64749907500006
Pd	10.49583732499997	10.49583732499997	18.35250092499994
Pd	18.35250092499994	10.49583732499997	14.50416267500003
Pd	14.50416267500003	14.50416267500003	6.64749907500006
Pd	10.49583732499997	18.35250092499994	14.50416267500003
Pd	18.35250092499994	14.50416267500003	10.49583732499997
Pd	10.49583732499997	14.50416267500003	18.35250092499994
Pd	14.50416267500003	6.64749907500006	14.50416267500003
Pd	14.50416267500003	18.35250092499994	10.49583732499997
Pd	10.49583732499997	6.64749907500006	10.49583732499997
Pd	14.50416267500003	10.49583732499997	18.35250092499994
Pd	6.64749907500006	14.50416267500003	14.50416267500003
Pd	10.49583732499997	10.49583732499997	6.64749907500006
Pd	6.64749907500006	10.49583732499997	10.49583732499997
Pd	18.35186165000007	16.45318382499994	12.50000000000000
Pd	18.35186165000007	8.54681617500006	12.50000000000000
Pd	6.64813834999993	16.45318382499994	12.50000000000000

Pd	6.64813834999993	8.54681617500006	12.50000000000000
Pd	12.50000000000000	18.35186165000007	16.45318382499994
Pd	12.50000000000000	18.35186165000007	8.54681617500006
Pd	12.50000000000000	6.64813834999993	16.45318382499994
Pd	12.50000000000000	6.64813834999993	8.54681617500006
Pd	16.45318382499994	12.50000000000000	18.35186165000007
Pd	16.45318382499994	12.50000000000000	6.64813834999993
Pd	8.54681617500006	12.50000000000000	6.64813834999993
Pd	8.54681617500006	12.50000000000000	18.35186165000007
Pd	18.35186165000007	12.50000000000000	16.45318382499994
Pd	12.50000000000000	16.45318382499994	6.64813834999993
Pd	8.54681617500006	18.35186165000007	12.50000000000000
Pd	18.35186165000007	12.50000000000000	8.54681617500006
Pd	12.50000000000000	16.45318382499994	18.35186165000007
Pd	16.45318382499994	6.64813834999993	12.50000000000000
Pd	16.45318382499994	18.35186165000007	12.50000000000000
Pd	8.54681617500006	6.64813834999993	12.50000000000000
Pd	12.50000000000000	8.54681617500006	18.35186165000007
Pd	6.64813834999993	12.50000000000000	16.45318382499994
Pd	12.50000000000000	8.54681617500006	6.64813834999993
Pd	6.64813834999993	12.50000000000000	8.54681617500006
Pd	20.20508457499997	14.47039159999992	12.50000000000000
Pd	20.20508457499997	10.52960840000008	12.50000000000000
Pd	4.79491542500003	14.47039159999992	12.50000000000000
Pd	4.79491542500003	10.52960840000008	12.50000000000000
Pd	12.50000000000000	20.20508457499997	14.47039159999992
Pd	12.50000000000000	20.20508457499997	10.52960840000008
Pd	12.50000000000000	4.79491542500003	14.47039159999992
Pd	12.50000000000000	4.79491542500003	10.52960840000008
Pd	14.47039159999992	12.50000000000000	20.20508457499997
Pd	14.47039159999992	12.50000000000000	4.79491542500003
Pd	10.52960840000008	12.50000000000000	4.79491542500003
Pd	10.52960840000008	12.50000000000000	20.20508457499997
Pd	20.20508457499997	12.50000000000000	14.47039159999992
Pd	12.50000000000000	14.47039159999992	4.79491542500003
Pd	10.52960840000008	20.20508457499997	12.50000000000000
Pd	20.20508457499997	12.50000000000000	10.52960840000008
Pd	12.50000000000000	14.47039159999992	20.20508457499997
Pd	14.47039159999992	4.79491542500003	12.50000000000000
Pd	14.47039159999992	20.20508457499997	12.50000000000000
Pd	10.52960840000008	4.79491542500003	12.50000000000000
Pd	12.50000000000000	10.52960840000008	20.20508457499997
Pd	4.79491542500003	12.50000000000000	14.47039159999992
Pd	12.50000000000000	10.52960840000008	4.79491542500003
Pd	4.79491542500003	12.50000000000000	10.52960840000008
Pd	18.45310629999997	16.51997124999998	16.51997124999998
Pd	18.45310629999997	8.48002875000003	8.48002875000003
Pd	6.54689370000004	16.51997124999998	8.48002875000003
Pd	6.54689370000004	8.48002875000003	16.51997124999998
Pd	16.51997124999998	18.45310629999997	16.51997124999998
Pd	8.48002875000003	18.45310629999997	8.48002875000003
Pd	8.48002875000003	6.54689370000004	16.51997124999998
Pd	16.51997124999998	6.54689370000004	8.48002875000003
Pd	16.51997124999998	16.51997124999998	18.45310629999997
Pd	16.51997124999998	8.48002875000003	6.54689370000004
Pd	8.48002875000003	16.51997124999998	6.54689370000004
Pd	8.48002875000003	8.48002875000003	18.45310629999997
Pd	18.45310629999997	8.48002875000003	16.51997124999998
Pd	16.51997124999998	16.51997124999998	6.54689370000004
Pd	8.48002875000003	18.45310629999997	16.51997124999998

Pd	18.45310629999997	16.51997124999998	8.48002875000003
Pd	8.48002875000003	16.51997124999998	18.45310629999997
Pd	16.51997124999998	6.54689370000004	16.51997124999998
Pd	16.51997124999998	18.45310629999997	8.48002875000003
Pd	8.48002875000003	6.54689370000004	8.48002875000003
Pd	16.51997124999998	8.48002875000003	18.45310629999997
Pd	6.54689370000004	16.51997124999998	16.51997124999998
Pd	8.48002875000003	8.48002875000003	6.54689370000004
Pd	6.54689370000004	8.48002875000003	8.48002875000003
Pd	18.42934429999996	18.42934429999996	14.57189195000002
Pd	18.42934429999996	6.57065570000004	10.42810804999998
Pd	6.57065570000004	18.42934429999996	10.42810804999998
Pd	6.57065570000004	6.57065570000004	14.57189195000002
Pd	14.51091278625716	18.41691364846084	18.41691364846084
Pd	10.42810804999998	18.42934429999996	6.57065570000004
Pd	10.42810804999998	6.57065570000004	18.42934429999996
Pd	14.57189195000002	6.57065570000004	6.57065570000004
Pd	18.42934429999996	14.57189195000002	18.42934429999996
Pd	18.42934429999996	10.42810804999998	6.57065570000004
Pd	6.57065570000004	14.57189195000002	6.57065570000004
Pd	6.57065570000004	10.42810804999998	18.42934429999996
Pd	18.42934429999996	10.42810804999998	18.42934429999996
Pd	14.57189195000002	18.42934429999996	6.57065570000004
Pd	6.57065570000004	18.42934429999996	14.57189195000002
Pd	18.42934429999996	14.57189195000002	6.57065570000004
Pd	10.48908721374286	18.41691364846084	18.41691364846084
Pd	18.42934429999996	6.57065570000004	14.57189195000002
Pd	18.42934429999996	18.42934429999996	10.42810804999998
Pd	6.57065570000004	6.57065570000004	10.42810804999998
Pd	14.57189195000002	6.57065570000004	18.42934429999996
Pd	6.57065570000004	14.57189195000002	18.42934429999996
Pd	10.42810804999998	6.57065570000004	6.57065570000004
Pd	6.57065570000004	10.42810804999998	6.57065570000004
Pd	20.31754120000002	16.47397082499999	14.53490004999995
Pd	20.31754120000002	8.52602917500001	10.46509995000005
Pd	4.68245879999998	16.47397082499999	10.46509995000005
Pd	4.68245879999998	8.52602917500001	14.53490004999995
Pd	14.51769057239433	20.29572483285161	16.46172530696801
Pd	10.46509995000005	20.31754120000002	8.52602917500001
Pd	10.46509995000005	4.68245879999998	16.47397082499999
Pd	14.53490004999995	4.68245879999998	8.52602917500001
Pd	16.47397082499999	14.53490004999995	20.31754120000002
Pd	16.47397082499999	10.46509995000005	4.68245879999998
Pd	8.52602917500001	14.53490004999995	4.68245879999998
Pd	8.52602917500001	10.46509995000005	20.31754120000002
Pd	20.31754120000002	10.46509995000005	16.47397082499999
Pd	14.53490004999995	16.47397082499999	4.68245879999998
Pd	8.52602917500001	20.31754120000002	14.53490004999995
Pd	20.31754120000002	14.53490004999995	8.52602917500001
Pd	10.48230942760567	16.46172530696801	20.29572483285161
Pd	16.47397082499999	4.68245879999998	14.53490004999995
Pd	16.47397082499999	20.31754120000002	10.46509995000005
Pd	8.52602917500001	4.68245879999998	10.46509995000005
Pd	14.53490004999995	8.52602917500001	20.31754120000002
Pd	4.68245879999998	14.53490004999995	16.47397082499999
Pd	10.46509995000005	8.52602917500001	4.68245879999998
Pd	4.68245879999998	10.46509995000005	8.52602917500001
Pd	4.68245879999998	8.52602917500001	10.46509995000005
Pd	4.68245879999998	16.47397082499999	14.53490004999995
Pd	20.31754120000002	8.52602917500001	14.53490004999995

Pd	20.31754120000002	16.47397082499999	10.46509995000005
Pd	10.46509995000005	4.68245879999998	8.52602917500001
Pd	14.53490004999995	4.68245879999998	16.47397082499999
Pd	14.53490004999995	20.31754120000002	8.52602917500001
Pd	10.48230942760567	20.29572483285161	16.46172530696801
Pd	8.52602917500001	10.46509995000005	4.68245879999998
Pd	8.52602917500001	14.53490004999995	20.31754120000002
Pd	16.47397082499999	10.46509995000005	20.31754120000002
Pd	16.47397082499999	14.53490004999995	4.68245879999998
Pd	4.68245879999998	14.53490004999995	8.52602917500001
Pd	10.46509995000005	8.52602917500001	20.31754120000002
Pd	16.47397082499999	4.68245879999998	10.46509995000005
Pd	4.68245879999998	10.46509995000005	16.47397082499999
Pd	14.53490004999995	8.52602917500001	4.68245879999998
Pd	8.52602917500001	20.31754120000002	10.46509995000005
Pd	8.52602917500001	4.68245879999998	14.53490004999995
Pd	16.47397082499999	20.31754120000002	14.53490004999995
Pd	10.46509995000005	16.47397082499999	4.68245879999998
Pd	20.31754120000002	10.46509995000005	8.52602917500001
Pd	14.51769057239433	16.46172530696801	20.29572483285161
Pd	20.31754120000002	14.53490004999995	16.47397082499999
Pd	20.24805837500008	12.50000000000000	18.34330087500007
Pd	20.24805837500008	12.50000000000000	6.65669912499993
Pd	4.75194162499992	12.50000000000000	6.65669912499993
Pd	4.75194162499992	12.50000000000000	18.34330087500007
Pd	18.34330087500007	20.24805837500008	12.50000000000000
Pd	6.65669912499993	20.24805837500008	12.50000000000000
Pd	6.65669912499993	4.75194162499992	12.50000000000000
Pd	18.34330087500007	4.75194162499992	12.50000000000000
Pd	12.50000000000000	18.49017175640736	20.44714643147156
Pd	12.50000000000000	6.65669912499993	4.75194162499992
Pd	12.50000000000000	18.34330087500007	4.75194162499992
Pd	12.50000000000000	6.65669912499993	20.24805837500008
Pd	20.24805837500008	6.65669912499993	12.50000000000000
Pd	18.34330087500007	12.50000000000000	4.75194162499992
Pd	12.50000000000000	20.44714643147156	18.49017175640736
Pd	20.24805837500008	18.34330087500007	12.50000000000000
Pd	6.65669912499993	12.50000000000000	20.24805837500008
Pd	12.50000000000000	4.75194162499992	18.34330087500007
Pd	12.50000000000000	20.24805837500008	6.65669912499993
Pd	12.50000000000000	4.75194162499992	6.65669912499993
Pd	18.34330087500007	12.50000000000000	20.24805837500008
Pd	4.75194162499992	18.34330087500007	12.50000000000000
Pd	6.65669912499993	12.50000000000000	4.75194162499992
Pd	4.75194162499992	6.65669912499993	12.50000000000000
Pd	22.19753022499997	12.50000000000000	12.50000000000000
Pd	2.80246977500003	12.50000000000000	12.50000000000000
Pd	12.50000000000000	22.19753022499997	12.50000000000000
Pd	12.50000000000000	2.80246977500003	12.50000000000000
Pd	12.50000000000000	12.50000000000000	22.19753022499997
Pd	12.50000000000000	12.50000000000000	2.80246977500003
Pd	22.17290320000007	14.42345097499995	14.42345097499995
Pd	22.17290320000007	10.57654902500005	10.57654902500005
Pd	2.82709679999993	14.42345097499995	10.57654902500005
Pd	2.82709679999993	10.57654902500005	14.42345097499995
Pd	14.41944896810612	22.16400887647420	14.41570932001986
Pd	10.57654902500005	22.17290320000007	10.57654902500005
Pd	10.57654902500005	2.82709679999993	14.42345097499995
Pd	14.42345097499995	2.82709679999993	10.57654902500005
Pd	14.41944896810612	14.41570932001986	22.16400887647420

Pd	14.42345097499995	10.57654902500005	2.82709679999993
Pd	10.57654902500005	14.42345097499995	2.82709679999993
Pd	10.57654902500005	10.57654902500005	22.17290320000007
Pd	22.17290320000007	10.57654902500005	14.42345097499995
Pd	14.42345097499995	14.42345097499995	2.82709679999993
Pd	10.58055103189387	22.16400887647420	14.41570932001986
Pd	22.17290320000007	14.42345097499995	10.57654902500005
Pd	10.58055103189387	14.41570932001986	22.16400887647420
Pd	14.42345097499995	2.82709679999993	14.42345097499995
Pd	14.42345097499995	22.17290320000007	10.57654902500005
Pd	10.57654902500005	2.82709679999993	10.57654902500005
Pd	14.42345097499995	10.57654902500005	22.17290320000007
Pd	2.82709679999993	14.42345097499995	14.42345097499995
Pd	10.57654902500005	10.57654902500005	2.82709679999993
Pd	2.82709679999993	10.57654902500005	10.57654902500005
Pd	22.12763370000008	16.35150097500002	12.50000000000000
Pd	22.12763370000008	8.64849902499998	12.50000000000000
Pd	2.87236629999992	16.35150097500002	12.50000000000000
Pd	2.87236629999992	8.64849902499998	12.50000000000000
Pd	12.50000000000000	22.17362528394081	16.37390306564940
Pd	12.50000000000000	22.12763370000008	8.64849902499998
Pd	12.50000000000000	2.87236629999992	16.35150097500002
Pd	12.50000000000000	2.87236629999992	8.64849902499998
Pd	16.35150097500002	12.50000000000000	22.12763370000008
Pd	16.35150097500002	12.50000000000000	2.87236629999992
Pd	8.64849902499998	12.50000000000000	2.87236629999992
Pd	8.64849902499998	12.50000000000000	22.12763370000008
Pd	22.12763370000008	12.50000000000000	16.35150097500002
Pd	12.50000000000000	16.35150097500002	2.87236629999992
Pd	8.64849902499998	22.12763370000008	12.50000000000000
Pd	22.12763370000008	12.50000000000000	8.64849902499998
Pd	12.50000000000000	16.37390306564940	22.17362528394081
Pd	16.35150097500002	2.87236629999992	12.50000000000000
Pd	16.35150097500002	22.12763370000008	12.50000000000000
Pd	8.64849902499998	2.87236629999992	12.50000000000000
Pd	12.50000000000000	8.64849902499998	22.12763370000008
Pd	2.87236629999992	12.50000000000000	16.35150097500002
Pd	12.50000000000000	8.64849902499998	2.87236629999992
Pd	2.87236629999992	12.50000000000000	8.64849902499998
C	12.50000000000000	20.51423811876282	20.51423811876282
C	12.50000000000000	22.50981309189233	18.31002105394973
C	12.50000000000000	18.31002105394973	22.50981309189233
O	12.50000000000000	21.34373716469467	21.34373716469467
O	12.50000000000000	23.45093281120073	19.01592546804719
O	12.50000000000000	19.01592546804719	23.45093281120073

4CO on 6Pd edge of Pd_{264} NP

Positions of 20 Pd centers were optimized together with the adsorbed CO molecules. The rest of Pd centers were fixed at their positions corresponding to the bare Pd_{264} NP.

-1310.863 eV

Pd	14.50839757161254	12.57059972153554	20.22510415210014
Pd	10.49160242838745	12.57059972153554	20.22510415210014
Pd	10.49076189597548	20.23114443456249	12.54495569204271
Pd	14.50923810402451	20.23114443456249	12.54495569204271
Pd	10.54470497402055	22.04851288933497	10.52200760438318
Pd	10.55501573884089	10.54727437621874	22.03176000709093
Pd	14.45529502597944	22.04851288933497	10.52200760438318

Pd	14.44498426115912	10.54727437621874	22.03176000709093
Pd	14.48949892054491	18.37073487162228	14.50143191435374
Pd	14.51617577749373	14.52510223599824	18.35368770727430
Pd	10.51050107945510	18.37073487162228	14.50143191435374
Pd	10.48382422250626	14.52510223599824	18.35368770727430
Pd	14.48190565030350	16.44586138775919	16.42658662617425
Pd	10.51809434969650	16.44586138775919	16.42658662617425
Pd	12.50000000000000	20.43761870011338	14.54122124316177
Pd	12.50000000000000	14.49476269077221	20.28014288376791
Pd	12.50000000000000	22.13757338204566	12.41724863118056
Pd	12.50000000000000	12.44323726491850	22.06424173089892
Pd	12.50000000000000	18.51205365203917	16.52785269527417
Pd	12.50000000000000	16.54110548807698	18.47540317909018
C	12.50000000000000	22.49596050168200	14.33895692140687
C	12.50000000000000	20.56616295032204	16.54159608020592
C	12.50000000000000	18.55248343841278	18.57162228163178
C	12.50000000000000	16.42643075947670	20.54896503228339
O	12.50000000000000	23.45407066819688	15.02376617112474
O	12.50000000000000	21.41671074937386	17.34854167223615
O	12.50000000000000	19.37304318177843	19.40883636307652
O	12.50000000000000	17.17053080656926	21.46023434136867

5CO on 6Pd edge of Pd_{264} NP

Positions of 20 Pd centers were optimized together with the adsorbed CO molecules. The rest of Pd centers were fixed at their positions corresponding to the bare Pd_{264} NP.

-1327.437 eV

Pd	14.50920973957557	12.54905019555752	20.22872134486535
Pd	10.49079026042443	12.54905019555752	20.22872134486535
Pd	10.49079026042443	20.22872134486535	12.54905019555752
Pd	14.50920973957557	20.22872134486535	12.54905019555752
Pd	10.54627906028749	22.04788956825198	10.52369675522116
Pd	10.54627906028749	10.52369675522116	22.04788956825198
Pd	14.45372093971251	22.04788956825198	10.52369675522116
Pd	14.45372093971251	10.52369675522116	22.04788956825198
Pd	14.49280494291945	18.36426316449492	14.50616969634104
Pd	14.49280494291945	14.50616969634104	18.36426316449492
Pd	10.50719505708056	18.36426316449492	14.50616969634104
Pd	10.50719505708056	14.50616969634104	18.36426316449492
Pd	14.47701326252096	16.43483022573500	16.43483022573500
Pd	10.52298673747904	16.43483022573500	16.43483022573500
Pd	12.50000000000000	20.43129340923923	14.54399676882625
Pd	12.50000000000000	14.54399676882625	20.43129340923923
Pd	12.50000000000000	22.13319785708433	12.42189271646540
Pd	12.50000000000000	12.42189271646540	22.13319785708433
Pd	12.50000000000000	18.50083071010164	16.52594731809974
Pd	12.50000000000000	16.52594731809974	18.50083071010164
C	12.50000000000000	22.49186810638684	14.34255671114872
C	12.50000000000000	20.56274452738784	16.53945840455095
C	12.50000000000000	18.55629074240960	18.55629074240960
C	12.50000000000000	16.53945840455095	20.56274452738784
C	12.50000000000000	14.34255671114872	22.49186810638684
O	12.50000000000000	23.44977281986926	15.02730648370254
O	12.50000000000000	21.41169382119838	17.34793862721379
O	12.50000000000000	19.38508038229307	19.38508038229307
O	12.50000000000000	17.34793862721379	21.41169382119838
O	12.50000000000000	15.02730648370254	23.44977281986926

6CO on 7Pd edge of Pd_{293} NP

Positions of 23 Pd centers were optimized together with the adsorbed CO molecules. The rest of Pd centers were fixed at their positions of the bare Pd_{293} NP.

```
-1383.932 eV
Pd   10.48400780345989   20.23188274417495   10.62018525647481
Pd   10.48400780345989   10.62018525647481   20.23188274417495
Pd   14.51599219654011   20.23188274417495   10.62018525647481
Pd   14.51599219654011   10.62018525647481   20.23188274417495
Pd   14.48236601602924   16.43731675393252   14.50018687878955
Pd   14.48236601602924   14.50018687878955   16.43731675393252
Pd   10.51763398397077   16.43731675393252   14.50018687878955
Pd   10.51763398397077   14.50018687878955   16.43731675393252
Pd   14.51875257919493   12.59989189876077   18.38830877017915
Pd   10.48124742080505   12.59989189876077   18.38830877017915
Pd   10.48124742080505   18.38830877017915   12.59989189876077
Pd   14.51875257919493   18.38830877017915   12.59989189876077
Pd   10.54150514295048   22.02625311677682   8.57362760227446
Pd   14.45849485704952   8.57362760227446   22.02625311677682
Pd   14.45849485704952   22.02625311677682   8.57362760227446
Pd   10.54150514295048   8.57362760227446   22.02625311677682
Pd   12.50000000000000   22.13749017550881   10.47532233774211
Pd   12.50000000000000   20.44540389683580   12.59488570618156
Pd   12.50000000000000   18.51612223447942   14.57174813791802
Pd   12.50000000000000   16.53609789380573   16.53609789380573
Pd   12.50000000000000   14.57174813791802   18.51612223447942
Pd   12.50000000000000   12.59488570618156   20.44540389683580
Pd   12.50000000000000   10.47532233774211   22.13749017550881
C   12.50000000000000   22.51863647400681   12.39031346941885
C   12.50000000000000   20.57130865960393   14.58932233444788
C   12.50000000000000   18.57363253600261   16.59126115423657
C   12.50000000000000   16.59126115423657   18.57363253600261
C   12.50000000000000   14.58932233444788   20.57130865960393
C   12.50000000000000   12.39031346941885   22.51863647400681
O   12.50000000000000   23.46735474706184   13.08599284624991
O   12.50000000000000   21.42561811582324   15.39286734566091
O   12.50000000000000   19.40378270294016   17.41805985853151
O   12.50000000000000   17.41805985853151   19.40378270294016
O   12.50000000000000   15.39286734566091   21.42561811582324
O   12.50000000000000   13.08599284624991   23.46735474706184
```

Slab with unit cell of 102 Pd atoms. Slab model of the edge terminating two (111) facets.

Four bottom layers (along Z axis) of Pd slab were fixed at their positions corresponding to Pd bulk (PBE, Pd-Pd distance of 279.7 pm). The rest of the slab was optimized.

```
-501.227 eV
Vectors:
    15.824     0.0000     0.000
     0.000     8.3919     0.000
     0.000     0.0000    27.973
Atomic coordinates:

Pd   0.00000000000000   0.00000000000000   0.00000000000000
Pd   3.95597959838125   0.00000000000000   0.00000000000000
Pd   7.91195919676250   0.00000000000000   0.00000000000000
Pd   11.86793879514375   0.00000000000000   0.00000000000000
Pd   0.00000000000000   2.79730000000002   0.00000000000000
Pd   3.95597959838125   2.79730000000002   0.00000000000000
Pd   7.91195919676250   2.79730000000002   0.00000000000000
```

Pd	11.86793879514375	2.79730000000002	0.00000000000000
Pd	0.00000000000000	5.59459999999998	0.00000000000000
Pd	3.95597959838125	5.59459999999998	0.00000000000000
Pd	7.91195919676250	5.59459999999998	0.00000000000000
Pd	11.86793879514375	5.59459999999998	0.00000000000000
Pd	1.97798979919063	1.39864999999998	1.39864999999992
Pd	5.93396939757188	1.39864999999998	1.39864999999992
Pd	9.88994899595312	1.39864999999998	1.39864999999992
Pd	13.84592859433437	1.39864999999998	1.39864999999992
Pd	1.97798979919063	4.19595000000000	1.39864999999992
Pd	5.93396939757188	4.19595000000000	1.39864999999992
Pd	9.88994899595312	4.19595000000000	1.39864999999992
Pd	13.84592859433437	4.19595000000000	1.39864999999992
Pd	1.97798979919063	6.99325000000002	1.39864999999992
Pd	5.93396939757188	6.99325000000002	1.39864999999992
Pd	9.88994899595312	6.99325000000002	1.39864999999992
Pd	13.84592859433437	6.99325000000002	1.39864999999992
Pd	0.00000000000000	0.00000000000000	2.79730000000004
Pd	3.95597959838125	0.00000000000000	2.79730000000004
Pd	7.91195919676250	0.00000000000000	2.79730000000004
Pd	11.86793879514375	0.00000000000000	2.79730000000004
Pd	0.00000000000000	2.79730000000002	2.79730000000004
Pd	3.95597959838125	2.79730000000002	2.79730000000004
Pd	7.91195919676250	2.79730000000002	2.79730000000004
Pd	11.86793879514375	2.79730000000002	2.79730000000004
Pd	0.00000000000000	5.59459999999998	2.79730000000004
Pd	3.95597959838125	5.59459999999998	2.79730000000004
Pd	7.91195919676250	5.59459999999998	2.79730000000004
Pd	11.86793879514375	5.59459999999998	2.79730000000004
Pd	1.97798979919063	1.39864999999998	4.19594999999996
Pd	5.93396939757188	1.39864999999998	4.19594999999996
Pd	9.88994899595312	1.39864999999998	4.19594999999996
Pd	13.84592859433437	1.39864999999998	4.19594999999996
Pd	1.97798979919063	4.19595000000000	4.19594999999996
Pd	5.93396939757188	4.19595000000000	4.19594999999996
Pd	9.88994899595312	4.19595000000000	4.19594999999996
Pd	13.84592859433437	4.19595000000000	4.19594999999996
Pd	1.97798979919063	6.99325000000002	4.19594999999996
Pd	5.93396939757188	6.99325000000002	4.19594999999996
Pd	9.88994899595312	6.99325000000002	4.19594999999996
Pd	13.84592859433437	6.99325000000002	4.19594999999996
Pd	0.00000000000000	0.00000000000000	5.58706361342694
Pd	3.95597959838125	0.00000000000000	5.59224668276066
Pd	7.91195919676250	0.00000000000000	5.58706361342694
Pd	11.86793879514375	0.00000000000000	5.59224668276066
Pd	0.00000000000000	2.79730000000002	5.58706361342694
Pd	3.95597959838125	2.79730000000002	5.59224668276066
Pd	7.91195919676250	2.79730000000002	5.58706361342694
Pd	11.86793879514375	2.79730000000002	5.59224668276066
Pd	0.00000000000000	5.59459999999998	5.58706361342694
Pd	3.95597959838125	5.59459999999998	5.59224668276066
Pd	7.91195919676250	5.59459999999998	5.58706361342694
Pd	11.86793879514375	5.59459999999998	5.59224668276066
Pd	1.94141917164977	1.39864999999998	6.99060502053310
Pd	5.97054002511273	1.39864999999998	6.99060502053310
Pd	9.85337836841227	1.39864999999998	6.99060502053310
Pd	13.88249922187523	1.39864999999998	6.99060502053310
Pd	1.94141917164977	4.19595000000000	6.99060502053310
Pd	5.97054002511273	4.19595000000000	6.99060502053310
Pd	9.85337836841227	4.19595000000000	6.99060502053310

```
Pd   13.88249922187523   4.19595000000000   6.99060502053310
Pd   1.94141917164977   6.99325000000002   6.99060502053310
Pd   5.97054002511273   6.99325000000002   6.99060502053310
Pd   9.85337836841227   6.99325000000002   6.99060502053310
Pd   13.88249922187523   6.99325000000002   6.99060502053310
Pd   0.00000000000000   0.00000000000000   8.46889967768126
Pd   3.95597959838125   0.00000000000000   8.31954201334869
Pd   7.91195919676250   0.00000000000000   8.46889967768126
Pd   11.86793879514375   0.00000000000000   8.31954201334869
Pd   0.00000000000000   2.79730000000002   8.46889967768126
Pd   3.95597959838125   2.79730000000002   8.31954201334869
Pd   7.91195919676250   2.79730000000002   8.46889967768126
Pd   11.86793879514375   2.79730000000002   8.31954201334869
Pd   0.00000000000000   5.59459999999998   8.46889967768126
Pd   3.95597959838125   5.59459999999998   8.31954201334869
Pd   7.91195919676250   5.59459999999998   8.46889967768126
Pd   11.86793879514375   5.59459999999998   8.31954201334869
Pd   1.94032265203260   1.39864999999998   9.77614684767020
Pd   5.97163654472990   1.39864999999998   9.77614684767020
Pd   9.85228184879510   1.39864999999998   9.77614684767020
Pd   13.88359574149240   1.39864999999998   9.77614684767020
Pd   1.94032265203260   4.19595000000000   9.77614684767020
Pd   5.97163654472990   4.19595000000000   9.77614684767020
Pd   9.85228184879510   4.19595000000000   9.77614684767020
Pd   13.88359574149240   4.19595000000000   9.77614684767020
Pd   1.94032265203260   6.99325000000002   9.77614684767020
Pd   5.97163654472990   6.99325000000002   9.77614684767020
Pd   9.85228184879510   6.99325000000002   9.77614684767020
Pd   13.88359574149240   6.99325000000002   9.77614684767020
Pd   3.95597959838125   0.00000000000000   11.00397405841057
Pd   11.86793879514375   0.00000000000000   11.00397405841057
Pd   3.95597959838125   2.79730000000002   11.00397405841057
Pd   11.86793879514375   2.79730000000002   11.00397405841057
Pd   3.95597959838125   5.59459999999998   11.00397405841057
Pd   11.86793879514375   5.59459999999998   11.00397405841057
```

Slab with unit cell of 68 Pd atoms

Four bottom layers of Pd slab were fixed at their positions corresponding to Pd bulk (PBE, Pd-Pd distance of 279.7 pm). The rest of the slab was optimized.

```
-334.274 eV
Vectors:
    15.824    0.0000    0.000
     0.000    5.5946    0.000
     0.000    0.0000   27.973
Atomic coordinates:
Pd   0.00000000000000   0.00000000000000   0.00000000000000
Pd   3.95597959838125   0.00000000000000   0.00000000000000
Pd   7.91195919676250   0.00000000000000   0.00000000000000
Pd   11.86793879514375   0.00000000000000   0.00000000000000
Pd   0.00000000000000   2.79730000000000   0.00000000000000
Pd   3.95597959838125   2.79730000000000   0.00000000000000
Pd   7.91195919676250   2.79730000000000   0.00000000000000
Pd   11.86793879514375   2.79730000000000   0.00000000000000
Pd   1.97798979919063   1.39865000000000   1.39864999999992
Pd   5.93396939757188   1.39865000000000   1.39864999999992
Pd   9.88994899595312   1.39865000000000   1.39864999999992
Pd   13.84592859433437   1.39865000000000   1.39864999999992
```

Pd	1.97798979919063	4.19595000000000	1.39864999999992
Pd	5.93396939757188	4.19595000000000	1.39864999999992
Pd	9.88994899595312	4.19595000000000	1.39864999999992
Pd	13.84592859433437	4.19595000000000	1.39864999999992
Pd	0.00000000000000	0.00000000000000	2.79730000000004
Pd	3.95597959838125	0.00000000000000	2.79730000000004
Pd	7.91195919676250	0.00000000000000	2.79730000000004
Pd	11.86793879514375	0.00000000000000	2.79730000000004
Pd	0.00000000000000	2.79730000000000	2.79730000000004
Pd	3.95597959838125	2.79730000000000	2.79730000000004
Pd	7.91195919676250	2.79730000000000	2.79730000000004
Pd	11.86793879514375	2.79730000000000	2.79730000000004
Pd	1.97798979919063	1.39865000000000	4.19594999999996
Pd	5.93396939757188	1.39865000000000	4.19594999999996
Pd	9.88994899595312	1.39865000000000	4.19594999999996
Pd	13.84592859433437	1.39865000000000	4.19594999999996
Pd	1.97798979919063	4.19595000000000	4.19594999999996
Pd	5.93396939757188	4.19595000000000	4.19594999999996
Pd	9.88994899595312	4.19595000000000	4.19594999999996
Pd	13.84592859433437	4.19595000000000	4.19594999999996
Pd	0.00000000000000	0.00000000000000	5.59460000000004
Pd	3.95597959838125	0.00000000000000	5.59460000000004
Pd	7.91195919676250	0.00000000000000	5.59460000000004
Pd	11.86793879514375	0.00000000000000	5.59460000000004
Pd	0.00000000000000	2.79730000000000	5.59460000000004
Pd	3.95597959838125	2.79730000000000	5.59460000000004
Pd	7.91195919676250	2.79730000000000	5.59460000000004
Pd	11.86793879514375	2.79730000000000	5.59460000000004
Pd	2.00659300725747	1.38685272066099	7.14033992879808
Pd	5.90536618950503	1.38685272066099	7.14033992879808
Pd	9.95812859327068	1.39451507850322	7.11810861488883
Pd	13.77774899701682	1.39451507850322	7.11810861488883
Pd	2.00659300725747	4.20774727933901	7.14033992879808
Pd	5.90536618950503	4.20774727933901	7.14033992879808
Pd	9.95812859327068	4.20008492149678	7.11810861488883
Pd	13.77774899701682	4.20008492149678	7.11810861488883
Pd	0.00000000000000	0.00000000000000	8.39190000000004
Pd	3.95597959838125	0.00000000000000	8.39190000000004
Pd	7.91195919676250	0.00000000000000	8.39190000000004
Pd	11.86793879514375	0.00000000000000	8.39190000000004
Pd	0.00000000000000	2.79730000000000	8.39190000000004
Pd	3.95597959838125	2.79730000000000	8.39190000000004
Pd	7.91195919676250	2.79730000000000	8.39190000000004
Pd	11.86793879514375	2.79730000000000	8.39190000000004
Pd	1.97798979919063	1.39865000000000	9.79054999999992
Pd	5.93396939757188	1.39865000000000	9.79054999999992
Pd	9.88994899595312	1.39865000000000	9.79054999999992
Pd	13.84592859433437	1.39865000000000	9.79054999999992
Pd	1.97798979919063	4.19595000000000	9.79054999999992
Pd	5.93396939757188	4.19595000000000	9.79054999999992
Pd	9.88994899595312	4.19595000000000	9.79054999999992
Pd	13.84592859433437	4.19595000000000	9.79054999999992
Pd	3.95597959838125	0.00000000000000	11.18920000000000
Pd	11.86793879514375	0.00000000000000	11.18920000000000
Pd	3.95597959838125	2.79730000000000	11.18920000000000
Pd	11.86793879514375	2.79730000000000	11.18920000000000

3CO on the slab of 102 Pd atoms

Four bottom layers of Pd slab were fixed at their positions corresponding to Pd bulk (PBE, Pd-Pd distance of 279.7 pm) . The rest of the slab was optimized together with adsorbed CO species.

```
-550.592 eV
C   3.95597959838125   1.39873910841166   12.68798008476327
C   3.95597959838125   4.19612711909130   12.68727793839649
C   3.95597959838125   6.99246201351267   12.68771617435092
O   3.95597959838125   1.39923384052454   13.85974121607091
O   3.95597959838125   4.19679129677581   13.85906941701825
O   3.95597959838125   6.99131197753581   13.85956606525673
Pd   0.00000000000000   0.00000000000000   0.00000000000000
Pd   3.95597959838125   0.00000000000000   0.00000000000000
Pd   7.91195919676250   0.00000000000000   0.00000000000000
Pd   11.86793879514375   0.00000000000000   0.00000000000000
Pd   0.00000000000000   2.79730000000002   0.00000000000000
Pd   3.95597959838125   2.79730000000002   0.00000000000000
Pd   7.91195919676250   2.79730000000002   0.00000000000000
Pd   11.86793879514375   2.79730000000002   0.00000000000000
Pd   0.00000000000000   5.59459999999998   0.00000000000000
Pd   3.95597959838125   5.59459999999998   0.00000000000000
Pd   7.91195919676250   5.59459999999998   0.00000000000000
Pd   11.86793879514375   5.59459999999998   0.00000000000000
Pd   1.97798979919063   1.39864999999998   1.39864999999992
Pd   5.93396939757188   1.39864999999998   1.39864999999992
Pd   9.88994899595312   1.39864999999998   1.39864999999992
Pd   13.84592859433437   1.39864999999998   1.39864999999992
Pd   1.97798979919063   4.19595000000000   1.39864999999992
Pd   5.93396939757188   4.19595000000000   1.39864999999992
Pd   9.88994899595312   4.19595000000000   1.39864999999992
Pd   13.84592859433437   4.19595000000000   1.39864999999992
Pd   1.97798979919063   6.99325000000002   1.39864999999992
Pd   5.93396939757188   6.99325000000002   1.39864999999992
Pd   9.88994899595312   6.99325000000002   1.39864999999992
Pd   13.84592859433437   6.99325000000002   1.39864999999992
Pd   0.00000000000000   0.00000000000000   2.79730000000004
Pd   3.95597959838125   0.00000000000000   2.79730000000004
Pd   7.91195919676250   0.00000000000000   2.79730000000004
Pd   11.86793879514375   0.00000000000000   2.79730000000004
Pd   0.00000000000000   2.79730000000002   2.79730000000004
Pd   3.95597959838125   2.79730000000002   2.79730000000004
Pd   7.91195919676250   2.79730000000002   2.79730000000004
Pd   11.86793879514375   2.79730000000002   2.79730000000004
Pd   0.00000000000000   5.59459999999998   2.79730000000004
Pd   3.95597959838125   5.59459999999998   2.79730000000004
Pd   7.91195919676250   5.59459999999998   2.79730000000004
Pd   11.86793879514375   5.59459999999998   2.79730000000004
Pd   1.97798979919063   1.39864999999998   4.19594999999996
Pd   5.93396939757188   1.39864999999998   4.19594999999996
Pd   9.88994899595312   1.39864999999998   4.19594999999996
Pd   13.84592859433437   1.39864999999998   4.19594999999996
Pd   1.97798979919063   4.19595000000000   4.19594999999996
Pd   5.93396939757188   4.19595000000000   4.19594999999996
Pd   9.88994899595312   4.19595000000000   4.19594999999996
Pd   13.84592859433437   4.19595000000000   4.19594999999996
Pd   1.97798979919063   6.99325000000002   4.19594999999996
Pd   5.93396939757188   6.99325000000002   4.19594999999996
Pd   9.88994899595312   6.99325000000002   4.19594999999996
Pd   13.84592859433437   6.99325000000002   4.19594999999996
Pd   0.01085605501624   8.39148745514929   5.59088247627385
Pd   3.95597959838125   -0.00056314280648   5.58332436988406
```

Pd	7.90110314174626	8.39148745514929	5.59088247627385
Pd	11.86793879514375	8.39159984858839	5.58876982528713
Pd	0.01081242249197	2.79684424986829	5.59080964479924
Pd	3.95597959838125	2.79679548857590	5.58336191221952
Pd	7.90114677427053	2.79684424986829	5.59080964479924
Pd	11.86793879514375	2.79695101616687	5.58876847205715
Pd	0.01072744737978	5.59417000411756	5.59073173825049
Pd	3.95597959838125	5.59406264050917	5.58340611504275
Pd	7.90123174938272	5.59417000411756	5.59073173825049
Pd	11.86793879514375	5.59430636694244	5.58881765147508
Pd	1.96999190316362	1.39795930664628	6.99878207361880
Pd	5.94196729359887	1.39795930664628	6.99878207361880
Pd	9.85558470538987	1.39801381822918	6.97913756174243
Pd	13.88029288489762	1.39801381822918	6.97913756174243
Pd	1.96985601750512	4.19528080577909	6.99884924442441
Pd	5.94210317925737	4.19528080577909	6.99884924442441
Pd	9.85561977569659	4.19533420658224	6.97911654777520
Pd	13.88025781459091	4.19533420658224	6.97911654777520
Pd	1.96988896929857	6.99252136244670	6.99882945706748
Pd	5.94207022746393	6.99252136244670	6.99882945706748
Pd	9.85558303859361	6.99270169693174	6.97912582177054
Pd	13.88029455169389	6.99270169693174	6.97912582177054
Pd	15.82164252065632	8.39092820401194	8.45479775987990
Pd	3.95597959838125	8.39111328540563	8.38956295152968
Pd	7.91423506963118	8.39092820401194	8.45479775987990
Pd	11.86793879514375	8.39130269856522	8.31298693188529
Pd	15.82159275842860	2.79644985531850	8.45466871537964
Pd	3.95597959838125	2.79647564124330	8.38944130041246
Pd	7.91428483185890	2.79644985531850	8.45466871537964
Pd	11.86793879514375	2.79670600314090	8.31300346808015
Pd	15.82160925050471	5.59364377274037	8.45469627875350
Pd	3.95597959838125	5.59382412656419	8.38934215219276
Pd	7.91426833978279	5.59364377274037	8.45469627875350
Pd	11.86793879514375	5.59400204867012	8.31302117912674
Pd	1.96364201117421	1.39753906968335	9.76054512390263
Pd	5.94831718558829	1.39753906968335	9.76054512390263
Pd	9.85401802337898	1.39798055809725	9.76363988805239
Pd	13.88185956690852	1.39798055809725	9.76363988805239
Pd	1.96366717472163	4.19480544449264	9.76066992663626
Pd	5.94829202204087	4.19480544449264	9.76066992663626
Pd	9.85396916435706	4.19529106251462	9.76371952842683
Pd	13.88190842593045	4.19529106251462	9.76371952842683
Pd	1.96371021472992	6.99217257651208	9.76066230612876
Pd	5.94824898203258	6.99217257651208	9.76066230612876
Pd	9.85399947856042	6.99256107385468	9.76365134328916
Pd	13.88187811172708	6.99256107385468	9.76365134328916
Pd	3.95597959838125	8.39133955858657	11.21988767539171
Pd	11.86793879514375	8.39130836069140	10.99806801630810
Pd	3.95597959838125	2.79710263482726	11.21899927319199
Pd	11.86793879514375	2.79676037321507	10.99812536022063
Pd	3.95597959838125	5.59432513490750	11.21842293046696
Pd	11.86793879514375	5.59402728143487	10.99813110942653

2CO on the slab of 68 Pd atoms

Four bottom layers of Pd slab were fixed at their positions corresponding to Pd bulk (PBE, Pd-Pd distance of 279.7 pm) . The rest of the slab was optimized together with adsorbed CO species.

-367.140 eV

C	3.95597959838125	1.39864688369075	12.68973787243290
C	3.95597959838125	4.19595311630925	12.68973787243290

O	3.95597959838125	1.39839134190173	13.86130083276922
O	3.95597959838125	4.19620865809827	13.86130083276922
Pd	0.00000000000000	0.00000000000000	0.00000000000000
Pd	3.95597959838125	0.00000000000000	0.00000000000000
Pd	7.91195919676250	0.00000000000000	0.00000000000000
Pd	11.86793879514375	0.00000000000000	0.00000000000000
Pd	0.00000000000000	2.79730000000000	0.00000000000000
Pd	3.95597959838125	2.79730000000000	0.00000000000000
Pd	7.91195919676250	2.79730000000000	0.00000000000000
Pd	11.86793879514375	2.79730000000000	0.00000000000000
Pd	1.97798979919063	1.39865000000000	1.39864999999992
Pd	5.93396939757188	1.39865000000000	1.39864999999992
Pd	9.88994899595312	1.39865000000000	1.39864999999992
Pd	13.84592859433437	1.39865000000000	1.39864999999992
Pd	1.97798979919063	4.19595000000000	1.39864999999992
Pd	5.93396939757188	4.19595000000000	1.39864999999992
Pd	9.88994899595312	4.19595000000000	1.39864999999992
Pd	13.84592859433437	4.19595000000000	1.39864999999992
Pd	0.00000000000000	0.00000000000000	2.79730000000004
Pd	3.95597959838125	0.00000000000000	2.79730000000004
Pd	7.91195919676250	0.00000000000000	2.79730000000004
Pd	11.86793879514375	0.00000000000000	2.79730000000004
Pd	0.00000000000000	2.79730000000000	2.79730000000004
Pd	3.95597959838125	2.79730000000000	2.79730000000004
Pd	7.91195919676250	2.79730000000000	2.79730000000004
Pd	11.86793879514375	2.79730000000000	2.79730000000004
Pd	1.97798979919063	1.39865000000000	4.19594999999996
Pd	5.93396939757188	1.39865000000000	4.19594999999996
Pd	9.88994899595312	1.39865000000000	4.19594999999996
Pd	13.84592859433437	1.39865000000000	4.19594999999996
Pd	1.97798979919063	4.19595000000000	4.19594999999996
Pd	5.93396939757188	4.19595000000000	4.19594999999996
Pd	9.88994899595312	4.19595000000000	4.19594999999996
Pd	13.84592859433437	4.19595000000000	4.19594999999996
Pd	0.00986220012189	0.00000000000000	5.59209881651756
Pd	3.95597959838125	0.00000000000000	5.58473995033978
Pd	7.90209699664062	0.00000000000000	5.59209881651756
Pd	11.86793879514375	0.00000000000000	5.59035673297029
Pd	0.00983227351319	2.79730000000000	5.59208501368366
Pd	3.95597959838125	2.79730000000000	5.58423212719100
Pd	7.90212692324931	2.79730000000000	5.59208501368366
Pd	11.86793879514375	2.79730000000000	5.59040385767927
Pd	1.97038921761190	1.39853477689334	6.99953852275502
Pd	5.94156997915060	1.39853477689334	6.99953852275502
Pd	9.85744913897216	1.39863274973253	6.98125146848925
Pd	13.87842845131534	1.39863274973253	6.98125146848925
Pd	1.97038921761190	4.19606522310666	6.99953852275502
Pd	5.94156997915060	4.19606522310666	6.99953852275502
Pd	9.85744913897216	4.19596725026747	6.98125146848925
Pd	13.87842845131534	4.19596725026747	6.98125146848925
Pd	-0.00399602401782	0.00000000000000	8.45767926085031
Pd	3.95597959838125	0.00000000000000	8.39197146294458
Pd	7.91595522078032	0.00000000000000	8.45767926085031
Pd	11.86793879514375	0.00000000000000	8.31810338658685
Pd	-0.00407271993723	2.79730000000000	8.45786169927699
Pd	3.95597959838125	2.79730000000000	8.39130304682159
Pd	7.91603191669973	2.79730000000000	8.45786169927699
Pd	11.86793879514375	2.79730000000000	8.31812367569225
Pd	1.96252713242186	1.39876681275011	9.75911378951326
Pd	5.94943206434064	1.39876681275011	9.75911378951326

Pd	9.85451796365457	1.39859675881431	9.76923806744383
Pd	13.88135962663293	1.39859675881431	9.76923806744383
Pd	1.96252713242186	4.19583318724989	9.75911378951326
Pd	5.94943206434064	4.19583318724989	9.75911378951326
Pd	9.85451796365457	4.19600324118569	9.76923806744383
Pd	13.88135962663293	4.19600324118569	9.76923806744383
Pd	3.95597959838125	0.00000000000000	11.21914120868164
Pd	11.86793879514375	0.00000000000000	11.00687278313683
Pd	3.95597959838125	2.79730000000000	11.21945745425142
Pd	11.86793879514375	2.79730000000000	11.00698371978026
